# Spatial Transcriptional Heterogeneity in the Infarct Core and Its Surrounding Regions Targeting Piezo1 Signals in Rats With Myocardial Ischemia‐Reperfusion Injury

**DOI:** 10.1002/mco2.70537

**Published:** 2026-01-02

**Authors:** Zhen Li, Fan Jiang, Yan Chen, Zhixiao Li, Yanqiong Wu, Zhigang He, Duozhi Wu, Hongbing Xiang

**Affiliations:** ^1^ Department of Anesthesiology and Pain Medicine Hubei Key Laboratory of Geriatric Anesthesia and Perioperative Brain Health Wuhan Clinical Research Center for Geriatric Anesthesia Tongji Hospital Tongji Medical College Huazhong University of Science and Technology Wuhan Hubei China; ^2^ Department of Anesthesiology Hainan General Hospital Hainan Affiliated Hospital of Hainan Medical University Haikou Hainan China; ^3^ Key Laboratory of Anesthesiology and Resuscitation (Huazhong University of Science and Technology) Ministry of Education Wuhan Hubei China

**Keywords:** myocardial ischemia reperfusion injury, Piezo1, single‐cell RNA sequencing, spatial transcriptomics

## Abstract

Myocardial ischemia‐reperfusion (MIR) injury is a major cause of cardiac dysfunction, but the spatial heterogeneity of its underlying molecular programs remains unclear. In this study, we applied Visium spatial transcriptomics to generate gene expression maps of rat left ventricles after MIR and identified distinct regional features. The border zones were enriched with phagosome‐related genes, incomplete infarct areas showed activation of MAPK, IL‐17, and osteoclast differentiation pathways, while the infarct cores were characterized by ferroptosis and mitophagy‐related genes. To further resolve the cellular basis, we integrated single‐cell RNA sequencing with RCTD deconvolution and found immune cell infiltration in infarct zones, neutrophil enrichment in incomplete infarct areas, and smooth muscle cell predominance in border zones. Both spatial and single‐cell analyses revealed altered expression of Piezo1, RyR2, MMP2, and SERCA2, which was further validated by Western blot and immunofluorescence co‐staining with ACTN2. Pseudotime analysis demonstrated selective enrichment and dynamic activation of Piezo1 in specific cardiomyocyte subclusters. Functional validation using a hypoxia/reoxygenation model confirmed that reoxygenation induced marked intracellular Ca^2+^ accumulation, which was attenuated by the Piezo1 inhibitor GsMTx4. Together, these findings delineate the spatial heterogeneity of MIR injury, identify Piezo1 as a key mediator of Ca^2+^ dysregulation, and suggest Piezo1 as a potential therapeutic target for myocardial protection.

## Graphical abstract

We employed spatially resolved transcriptomics to visualize and molecularly characterize the spatial distribution of gene expression profiles in the rats’ left ventricles following MIR injury. Further experiments indicated that cardiogenic Piezo1‐mediated calcium overload exacerbates MIR through the activation of matrix metalloproteinase 2‐ryanodine receptor 2/sarcoplasmic/endoplasmic reticulum Ca^2+^‐ATPase 2 signaling.

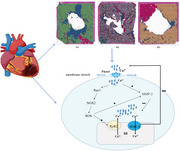

## 1 Introduction

The prompt and effective reperfusion of blood flow can alleviate myocardial ischemia injury, minimize infarct size, enhance ventricular function, and decrease acute mortality rates in patients with acute myocardial infarction (MI), which remains a leading cause of mortality globally [1, 2]. However, it is important to note that the restoration of blood flow may also lead to undesired myocardial ischemia‐reperfusion (MIR) injury [3, 4]. These consequences often manifest at the cellular and molecular level as cardiomyocyte necrosis, endothelial dysfunction, altered calcium handling, metabolic and oxidative stress, pH paradox, and inflammation [5]. Pharmacological interventions targeting these pathophysiological changes have shown clinical efficacy by normalizing metabolism, mitigating oxidative stress, and reducing inflammation. Nevertheless, these therapeutic agents lack specificity for particular tissues or tissue‐wide gene expression heterogeneity following MIR injury, which may result in unpredictable adverse effects [6, 7]. Therefore, a consensus treatment approach to minimize the adverse outcomes of MIR injury remains elusive. A more localized and targeted strategy is likely to hold the key to developing more effective therapies for MIR injury.

The intricate and diverse nature of participating cell types and subtypes, along with their complex interrelationships, has necessitated the utilization of single‐cell RNA sequencing to gain a deeper understanding of the mechanisms underlying the injury response [8, 9, 10]. Although scRNA‐seq studies offer valuable insights, they ultimately lack the spatial information necessary to establish a correlation between transcriptional state dynamics and MIR injury [11]. In contrast, spatial transcriptomics enhances scRNA‐seq by preserving 2D positional information of cells while providing expression data [12]. A recent study employed imaging mass cytometry to analyze the spatiotemporal heterogeneity of cellular phenotypes in the mouse heart following MIR injury. The study utilized heart sections collected from 12 distinct cardiac regions across various time points [13]. However, there is limited understanding regarding the crucial spatiotemporal sequencing of molecular events that underlie the processes of infarction and incomplete infarction due to an inaccurate distinction between infarct and incomplete infarct zones.

During myocardial ischaemia‐reperfusion (I/R), cardiomyocytes swell and may rupture, altering membrane tension [14, 15]. Early interstitial edema and cytoskeletal remodeling increase tissue stiffness and create regional mechanical heterogeneity [16]. Mechanical cues reshape intracellular Ca^2+^ handling and downstream signaling in cardiomyocytes [17]. Piezo1, a mechanosensitive ion channel, is expressed in cardiomyocytes and senses force generated by contraction–relaxation cycles [18, 19], converting membrane tension and shear into Ca^2+^ and ROS signals relevant to cardiac function and vascular remodeling [20, 21, 22, 23]. In I/R settings, Piezo1 expression is upregulated; cardiomyocyte‐specific deletion reduces infarct size and preserves mitochondrial homeostasis [24]. Pharmacological inhibition with GsMTx4‐D alleviates myocardial injury in mice and in vitro, consistent with reduced Ca^2+^ overload and PANoptosis‐related signaling [25]. The spatial distribution and expression dynamics of Piezo1 in injured myocardium, however, remain insufficiently defined.

Therefore, we apply spatial transcriptomics to resolve transcriptional heterogeneity across infarct and peri‐infarct territories, integrate single‐cell RNA‐seq by RCTD for cell‐type mapping, and delineate infarct‐core boundaries. Pseudotime analysis characterizes cardiomyocyte state transitions. A Piezo1‐high cardiomyocyte subset emerges from this framework; enrichment analyses indicate its association with key signaling features relevant to I/R injury.

## 2 Results

### 2.1 Spatial Transcriptome Profiling of Ventricle Samples Upon MIR Injury

**FIGURE 1:**
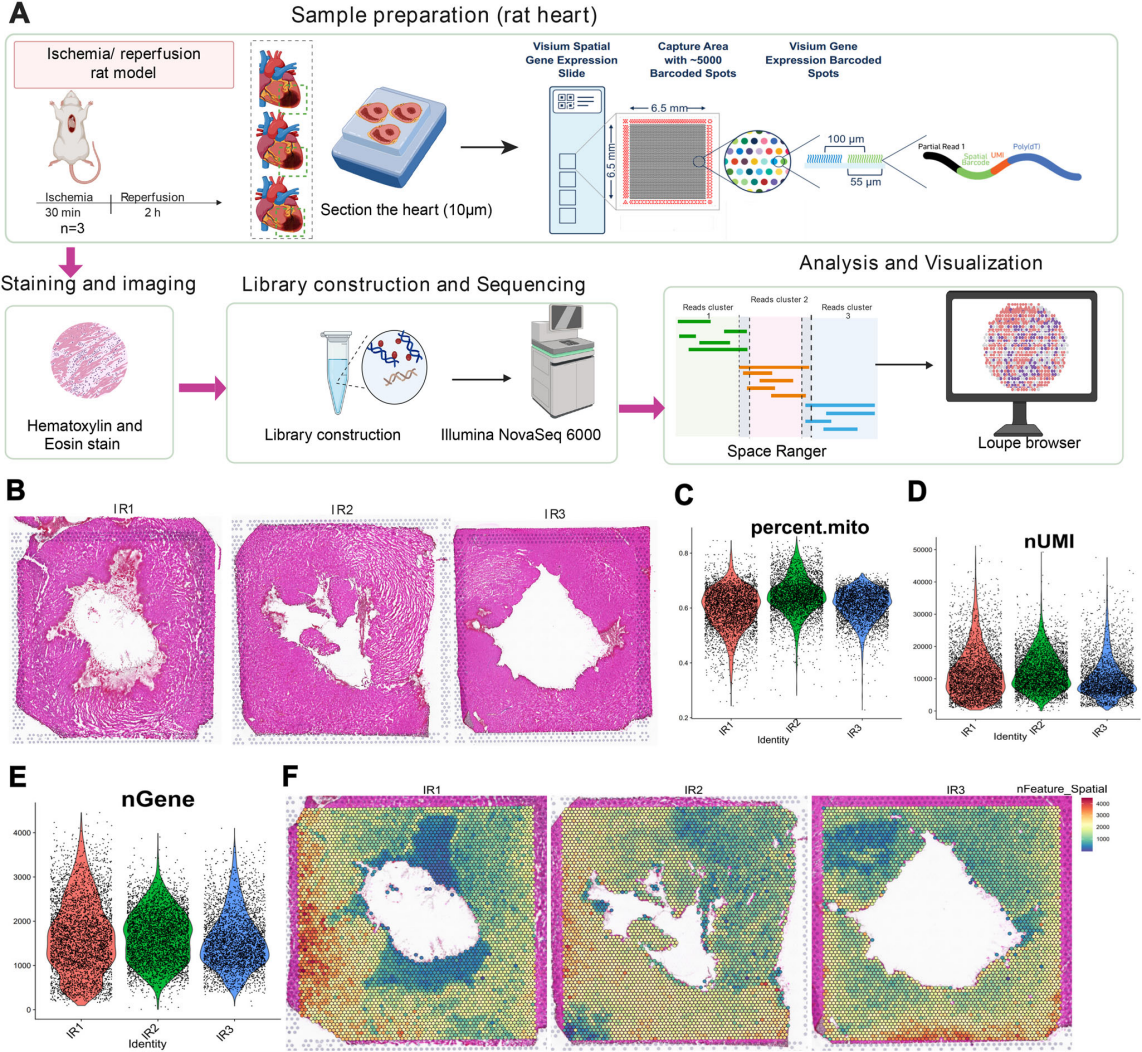
A spatial transcriptomic atlas of rat hearts after myocardial ischemia‐reperfusion (MIR) injury. (A) An overview of the experimental workflow. (B) An annotated bright‐field image of an H&E‐stained tissue section. (C) Distribution of mitochondrial genes in three hearts. (D) Distribution of UMI in hearts. (E) Distribution of all expressed gene numbers in hearts. (F) Spatial distributions of the numbers of expressed genes in three hearts after MIR injury. UMI, Unique Molecular Index.

Using the 10× Genomics Visium platform, we conducted spatial transcriptomics (ST) on three left ventricle samples obtained from rats following MIR injury (Figure 1A). As depicted in Figure 1A, transcriptomes were sequenced from a total of 4214, 4143, and 3600 spots for each sample, respectively. After normalization, the mean reads per spot reached values of 90,994, 75,166, and 92,027. The data underwent subsequent steps, including batch effect correction across samples and dimensionality reduction to facilitate further analysis. Moreover, hematoxylin and Eosin (H&E)‐stained images from each sample were meticulously examined by the study pathologist, who annotated morphological regions as shown in Figure 1B. The percentage of reads mapped to mitochondria, UMI (Unique Molecular Index), and the number of genes are shown in Figure 1C–E. Spatial distribution patterns of gene numbers are presented in Figure 1F.

### 2.2 Gene Profiling Was Heterogeneous Throughout the Left Ventricle Following MIR Injury

**FIGURE 2:**
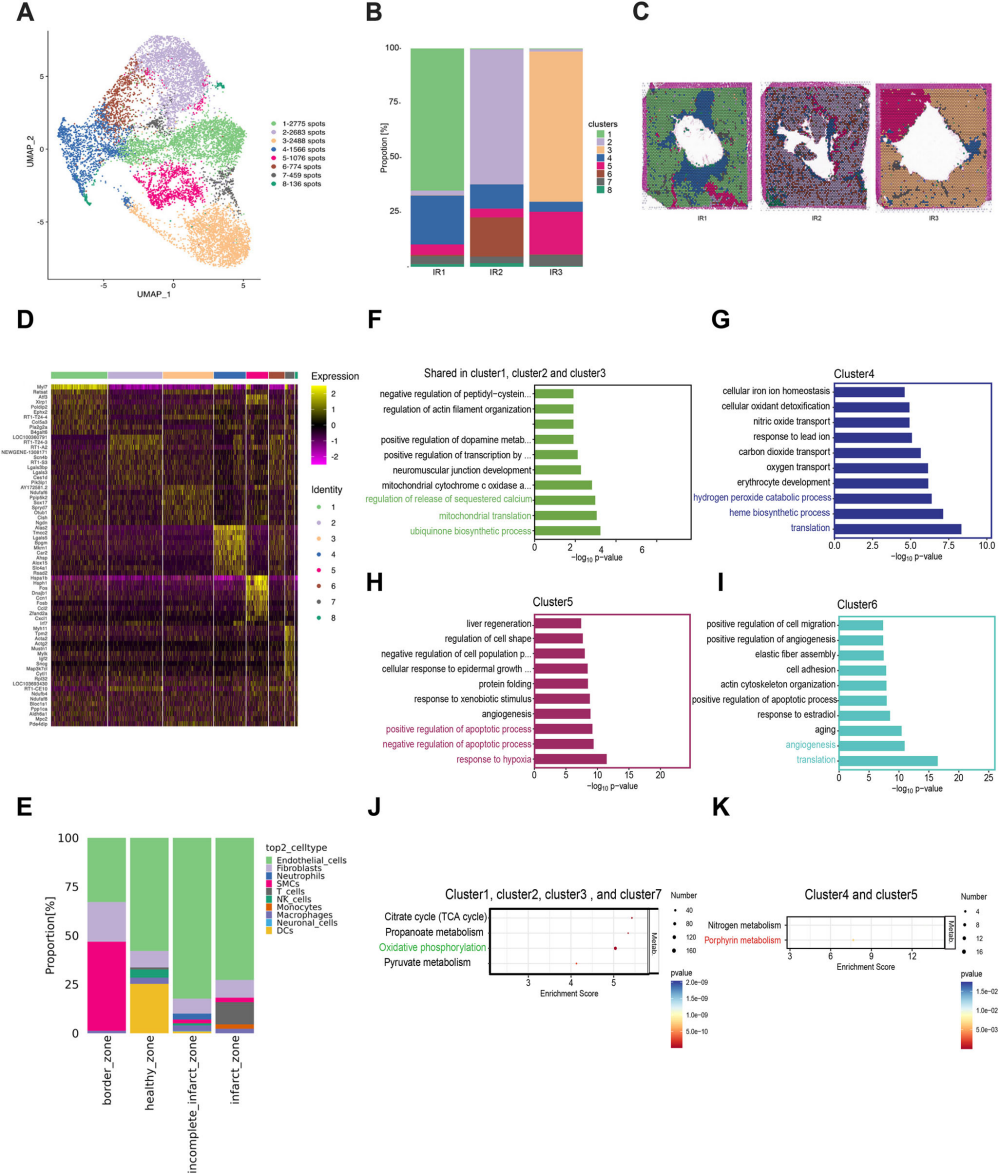
Spatial heterogeneity and functional architecture of myocardial regions after MIR injury. (A) UMAP plots classify genes into eight clusters based on their overall gene expression relationship among the 11,957 spots with 2410 genes. Different gene clusters are color‐coded. (B) Spatial distribution of all clusters in three samples. UMAP, uniform manifold approximation and projection. (C) The proportion of each cluster in each heart after MIR injury. (D) Heatmap of the top 10 genes of all clusters in three hearts after MIR injury. (E) Proportions of non‐cardiomyocyte cell types across distinct myocardial regions based on RCTD mapping of scRNA‐seq profiles onto spatial transcriptomic data. (F) The top 10 biological processes shared in the non‐infarcted heart that are resistant to MIR injury. (G) The top 10 biological processes identified in infarct zones (Cluster 4). (H) The top 10 biological processes found in incomplete infarct zones (Cluster 5). (I) The top 10 biological processes in border areas surrounding the infarct zones. (J) The energy metabolism process is shared among the distal non‐infarcted areas after MIR injury. (K) The energy metabolism process is shared between the infarct zones and the incomplete infarct zones.

After identifying and annotating infarct areas and incomplete infarct areas, we conducted unsupervised Louvain clustering to uncover the spatial heterogeneity in ventricular expression profiles. We generated eight Louvain clusters among aggregated spots that could be divided into five major clusters (Figure 2A). The genes in Cluster 1, Cluster 2, and Cluster 3, respectively, represent the major non‐infarcted heart genes resistant to MIR injury in three rats (Figure 2B,C). Remarkably, when mapping the Cluster ed spots back to their original morphological spatial locations, they corresponded precisely to defined anatomical regions. The fourth Cluster comprised genes expressed within the infarct zones, the fifth Cluster consisted of genes expressed within incomplete infarct areas, and the seventh Cluster encompassed genes expressed in border regions.

To further classify these clusters, we subsequently characterized the major clusters through differential gene expression analysis. The top 10 specifically expressed genes in each Cluster are presented in Figure 2D. Marker genes shared among Cluster 1, Cluster 2, and Cluster 3 included genes involved in the ribosome (Mrpl13, Mrpl4, Mrpl20, and Mrps18c) and TGF‐β signaling pathway (Hjv, Cul1, and Mapk3), which have been demonstrated to possess anti‐inflammatory effects and promote angiogenesis during myocardial ischemia‐reperfusion [26, 27]. Top marker genes enriched in Cluster 4 included Alas2, Tmcc2, Lgals5, Bpgm, Mkrn1, Car2, Ahsp, Alox15, Slc4a1, and Rsad2. These genes are primarily involved in ferroptosis and focal adhesion. Additionally, the top marker genes in Cluster 5 (Hspa1b, Hsph1, Fos, Dnajb1, Atf3, Ccn1, Fosb, Ccl2, Zfand2a, and Cxcl1) were found to be associated with the mitogen‐activated protein kinase (MAPK) signaling pathway as well as focal adhesion and osteoclast differentiation pathways. Marker genes enriched in Cluster 7 are implicated in focal adhesion and the regulation of actin cytoskeleton dynamics (Tpm2, Actg2, and Mylk). Cluster 8, in contrast, represented only a very small fraction of spots with dispersed and low‐quality gene expression, and was therefore not included in subsequent biological interpretation.

Notably, the ventricular area where the marker genes in Clusters 4, 5, and 7 reside corresponds precisely to the infarct zones, incomplete infarction areas, and border areas, respectively. These findings prompted us to annotate Cluster 4 as genes associated with infarct zones (pan‐necrosis), Cluster 5 as genes related to incomplete infarction areas (scattered necrotic regions interspersed with preserved tissue), and Cluster 7 as border region genes due to their histological characteristics that remained unchanged even in proximity to the infarct zones and areas of incomplete infarction. To further dissect the cellular composition across these anatomical regions, we integrated single‐cell transcriptomic data with spatial transcriptomics using RCTD. Because cardiomyocytes constitute the vast majority of the ventricular tissue, they were excluded from the proportional display to better visualize non‐cardiomyocyte differences. RCTD‐based deconvolution revealed that the infarct zone was characterized by an increase in immune cells, particularly monocytes, macrophages, and T cells; the incomplete infarct zone exhibited evident neutrophil infiltration together with a notable fraction of smooth muscle cells (SMCs); and the border zone was marked by a pronounced increase in SMCs, accompanied by a proportion of fibroblasts (Figure 2E). These results suggest that regional vascular responses and the onset of inflammation are already evident at the early reperfusion stage.

### 2.3 Functional and Metabolic Architecture Was Heterogeneous Throughout the Left Ventricle Following MIR Injury

Subsequently, we conducted GO analysis to investigate the biological function of each cluster, revealing distinct attributes unique to each cluster. The biological processes shared in the non‐infarcted heart (Cluster 1, Cluster 2, and Cluster 3) are mainly linked to the ubiquinone biosynthetic process (Coq10a and Coq7), mitochondrial translation (Mrpl13, Mrpl20, and Mrpl4), and the regulation of release of sequestered calcium (Dhrs7c and Gsto1) (Figure 2F). The marker genes associated with translation, heme biosynthesis, and the hydrogen peroxide degradation process were significantly enriched in the infarct zones, whereas marker genes related to hypoxia response and regulation of the apoptotic process were highly enriched in the incomplete infarct areas (Figure 2G,H). In border regions, enrichment included translation and angiogenesis‐related terms (Figure 2I). KEGG analysis revealed that the pathways activated in the distal non‐infarcted regions (including Clusters 1, 2, 3, and 7) are primarily associated with oxidative phosphorylation (Figure 2J). Interestingly, Clusters 4 and 5 showed enrichment in porphyrin metabolism, suggesting a potential shared metabolic feature between these regions (Figure 2K) (Cluster 4: *p* = 0.0007; Cluster 5: *p* = 0.003).

### 2.4 Gene Expression Differences in the Incomplete Infarction Areas and the Border Regions

**FIGURE 3:**
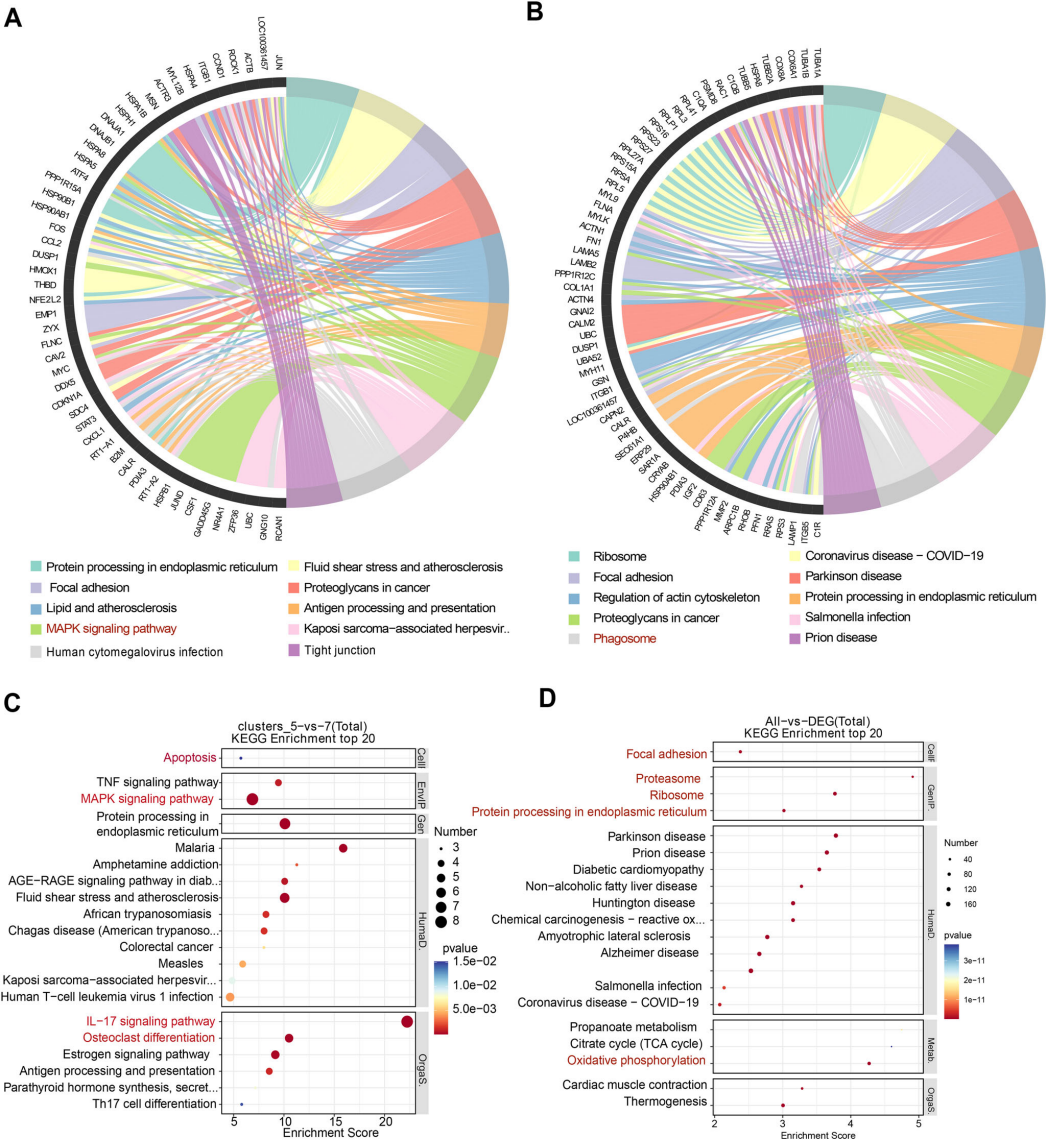
Gene expression differences in the incomplete infarction areas and the border regions. (A) Top 10 KEGG pathways enrichment analysis of marker genes in the incomplete infarction areas. (B) Enrichment analysis of the top 10 KEGG pathways in the border areas. (C) Enrichment analysis of the top 10 KEGG pathways for significantly differentially expressed genes between the incomplete infarction areas and border regions. (D) Top 20 KEGG pathways enrichment analysis of marker genes in the entire ischemic cardiac apex.

We aimed to further investigate the functional disparities in gene expression between the areas of incomplete infarction and their adjacent border regions, as well as elucidate how signals from the incomplete infarction stimulate the neighboring ventricle. KEGG pathway enrichment analysis revealed that genes in incomplete infarction areas were significantly enriched for MAPK signaling pathways (Figure 3A), while genes in the border regions showed enrichment for focal adhesion and phagosomal factors (Figure 3B). Subsequently, DEGs between incomplete infarction areas and border regions were identified. A total of 230 DEGs were extracted using a cutoff criterion of *p*‐value less than 0.05 and fold change greater than 1.5, among which 135 genes were upregulated in the incomplete infarction areas (Figure 3C). Notably, there was a marked upregulation of genes involved in MAPK signaling pathways (Hspa1b, Hspb1, Fos, Hspa8, Jund, Dusp1, Jun, and Gadd45g), IL‐17 signaling pathways (Fos, Ccl2, Hsp90ab1, Fosb, Jund, Jun, S100a8, and S100a9), as well as osteoclast differentiation signaling pathways (Fos, Junb, Fosb, Jund, and Jun) in the incomplete infarction areas (Figure 3C). Furthermore, the expression levels of apoptosis‐related pathway signaling genes (Fos, Jun, and Gadd45g) were found to be profoundly upregulated in the incomplete infarction areas.

We also conducted KEGG analysis of genes from the eight clusters (Figure 3D) to dissect the regulatory pathways involved in the ischemic process. The predominant pathways in the entire ischemic cardiac apex primarily pertain to altered genetic information processing, including ribosome‐related pathways, protein processing in the endoplasmic reticulum (ER), and proteasome‐related pathways. Additionally, they involve cellular processes related to focal adhesion and cellular metabolism associated with oxidative phosphorylation.

### 2.5 Disparities and Resemblances in Gene Expression Between the Infarct Zones and the Incomplete Infarct Areas

**FIGURE 4:**
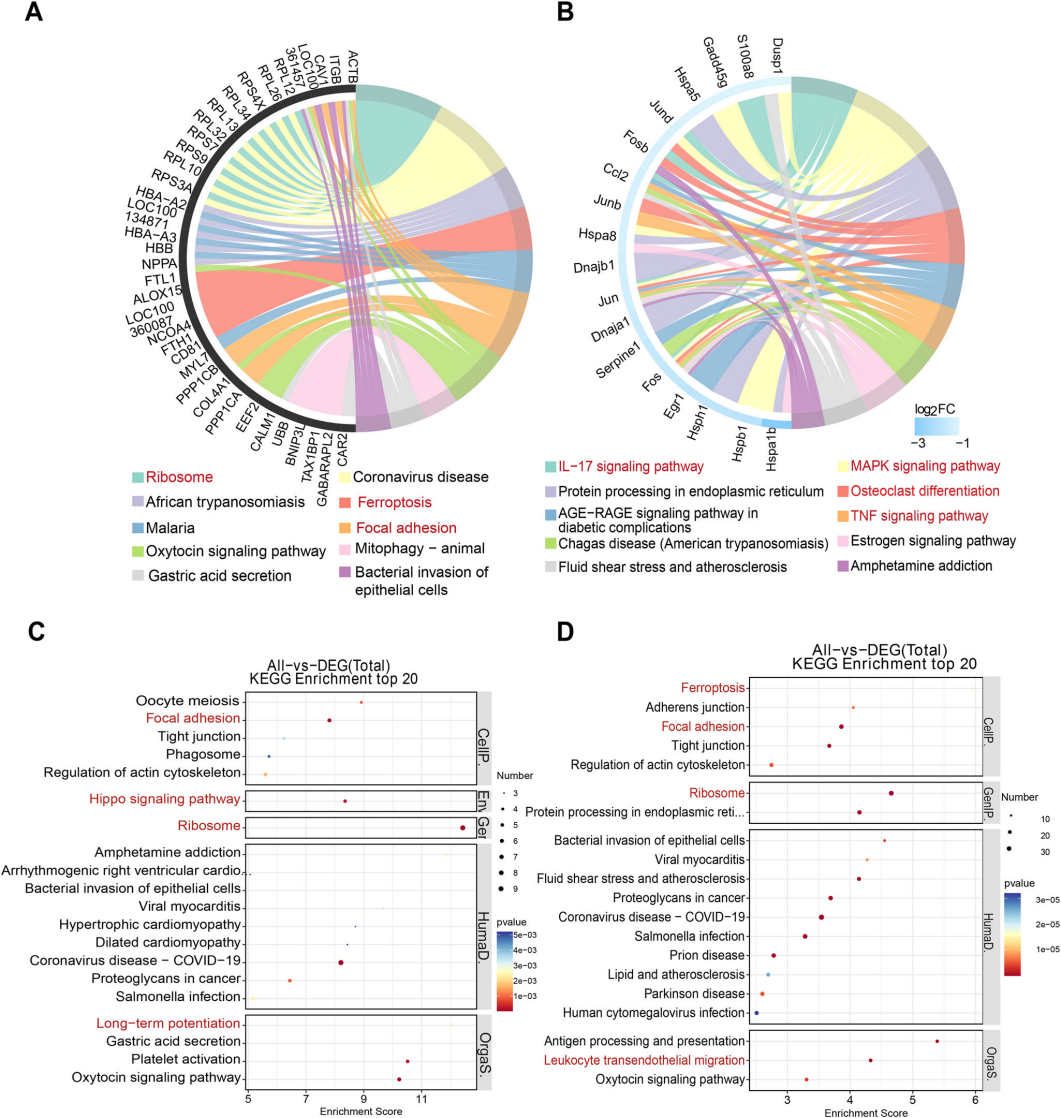
Differences and similarities in gene expression between the infarct zones and the incomplete infarct areas. (A) Top 10 KEGG pathways enrichment analysis of marker genes from infarct zones. (B) Top 10 KEGG pathways enrichment analysis of significantly differentially expressed genes between the infarct zones and the incomplete infarct areas. (C) Top 20 KEGG pathways enrichment analysis of marker genes shared between the infarct and the incomplete infarct areas. (D) Top 20 KEGG pathway enrichment analysis of the marker genes in the infarct and the incomplete infarct areas.

To gain insight into alterations in gene expression concerning the pathophysiology of MIR injury, we focused on the infarct areas (Cluster 4) and the incomplete infarct areas (Cluster 5) of the ventricle. First, we conducted KEGG analysis on marker genes from Cluster 4 to investigate the regulatory pathways associated with the process of infarction. KEGG pathway enrichment analysis of the infarction areas shows that they are enriched for ferroptosis‐related pathways (Ftl1, Alox15, LOC100360087, Ncoa4, and Fth1), ribosome‐related pathways, and focal adhesion‐related pathways (Figure 4A). Next, we compared gene expression between Clusters 4 and 5 to explore regulatory differences between the complete and incomplete infarct process. We identified a total of 68 DEGs using a cutoff standard of *p*‐value < 0.05 and FC > 1.5; among them, 45 genes were upregulated in the incomplete infarction areas. The KEGG pathway enrichment analysis of these DEGs showed their enrichment in MAPK signaling pathway (Fos, Jun, Jund, Hspa1b, Hspb1, Hspa8, Gadd45g, and Dusp1), IL−17 signaling pathway (Fos, Jun, Ccl2, Fosb, Jund, and S100a8), osteoclast differentiation signaling pathways genes (Fos, Junb, Fosb, Jund, and Jun), and tumor necrosis factor (TNF) signaling pathway (Fos, Jun, Ccl2, and Junb) (Figure 4B).

To further explore the shared pathological regulatory pathways between infarction and incomplete infarction processes, we analyzed marker genes that are common to both infarcted and incompletely infarcted areas. KEGG pathway enrichment analysis revealed significant enrichments in ribosome‐related pathways, focal adhesion‐related pathways, and the Hippo signaling pathway (Actb, Ppp1cb, LOC100361457, Ywhah, and Ppp1ca) (Figure 4C). We also analyzed the expression of marker genes in both infarcted and incompletely infarcted areas, aiming to unravel critical signaling involved in the onset and progression of acute myocardial infarction. Not surprisingly, KEGG pathway enrichment analysis revealed that genes within Clusters 4 and 5 were significantly enriched for ribosome‐related pathways, focal adhesion‐related pathways, leukocyte transendothelial migration‐related pathways (Myl7, Actb, Itgb1, LOC100361457, Rock1, Gnai3, Myl12b, Msn, Actn4, Rhoa, Rock2, Cd99, Mmp2, Vcl, Cdh5, Cldn5, Myl12a, Rac1, and Gnai2), as well as ferroptosis‐related pathways (Figure 4D). Collectively, our findings suggest that genes in the infarct and the incomplete infarct areas engage in distinct patterns of pathological regulatory pathways, further shaping their unique biologies during the process of infarction and incomplete infarction.

### 2.6 Comparison of the Infarct and Non‐Infarct Zones Gene Expression

**FIGURE 5:**
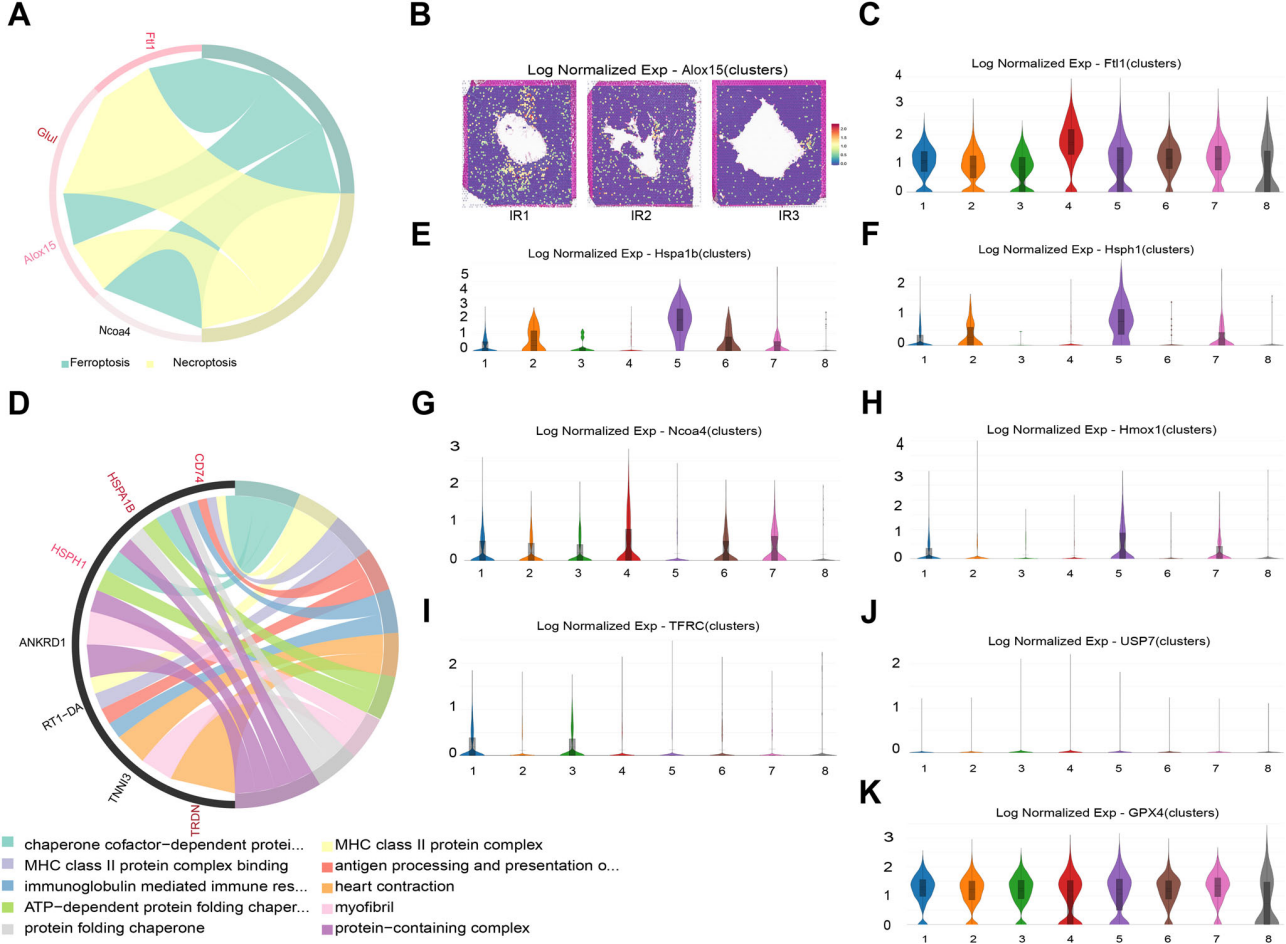
Comparison of the infarcted and non‐infarcted zones gene expression.(A) Top 10 KEGG pathway enrichment analysis of the upregulated genes in the infarcted zones. (B) Distributions of Alox15 expression in three hearts. (C) The expression of Ftl1 among clusters. (D) The downregulated genes in the infarcted zones. (E and F) Hspa1b and Hsph1 were downregulated within the infarcted zones (Cluster 4), but conversely upregulated within incomplete infarct areas (Cluster 5). (G–K) The expression of ferroptosis‐related genes among groups.

To elucidate signaling patterns and epigenetic regulation following myocardial infarction reperfusion, we compared gene expression of the infarct zones (Cluster 4) with the non‐infarct zones (all clusters except Cluster 4) after MIR injury. We identified 65 differentially expressed genes, of which 44 are upregulated in the infarct zones. As anticipated, KEGG pathway enrichment analysis of these upregulated genes revealed their enrichment for ferroptosis‐related pathways (Ftl1, Alox15, and Ncoa4) and necroptosis‐related pathways (Ftl1, Alox15, and Glul) (Figure 5A). Spatial maps showed infarct‐restricted upregulation of Alox15 across all hearts (IR1–IR3), with the weaker visual impression in IR2/IR3 attributable to smaller infarct extents (Figure 5B). Violin plots confirmed that Ftl1, another ferroptosis‐related gene, exhibited its highest expression in Cluster  4, which corresponds to the infarct zone (Figure 5C). In contrast, mitochondrial genes Tnni3, Ankrd1, CD74, triadin, and RT1‐Da are downregulated in the infarct zones (Figure 5D). Of note, Hspa1b and Hsph1 were downregulated within the infarcted zones (Cluster 4), but conversely upregulated within incomplete infarct areas (Cluster 5) (Figure 5E,F).

Our findings indicate that Ncoa4 expression was specifically upregulated in infarcted zones, while HMOX‐1 was upregulated in incomplete infarct areas (Figure 5G,H). In contrast, TFRC and USP7 remained unchanged (Figure 5I,J), suggesting that ferritinophagy is the primary cause of iron overload during MI/R Injury. However, there was no significant change in GPX4 expression between groups (Figure 5K).

### 2.7 Deficiency in Macroautophagy and Excessive Mitophagy may Aggravate MIR Injury

**FIGURE 6:**
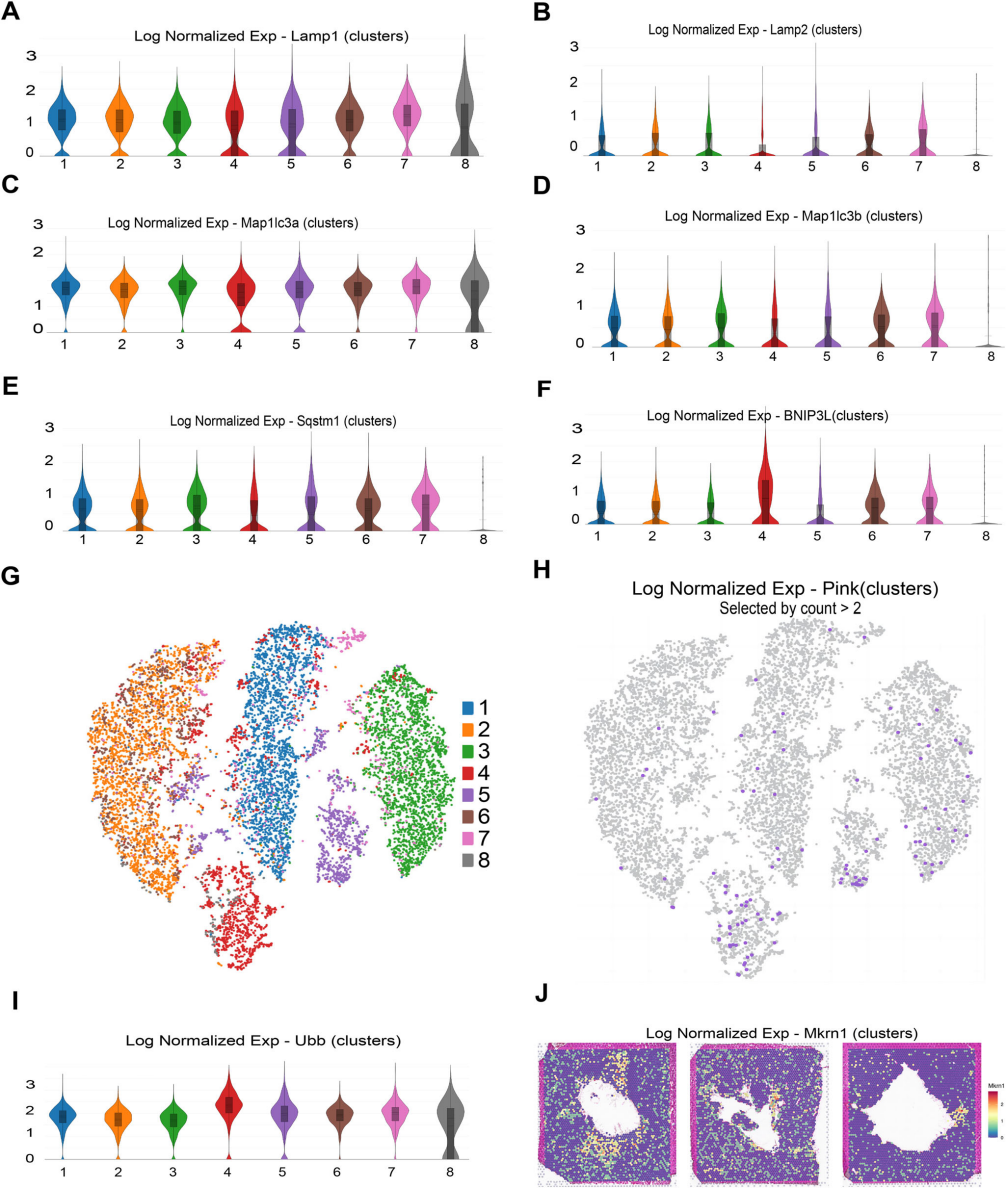
A deficiency in macroautophagy and excessive mitophagy in the infarcted areas. (A–E) Expression of genes related to macroautophagy. (F) The expression of the mitophagy‐related gene (BNIPL3) was increased within the infarcted areas. (G) A merged tSNE visualization classification of the clusters in rat hearts. (H) The expression of the mitophagy‐related gene (pink) was increased within the infarcted areas. (I and J) Ubiquitin and E3 ubiquitin ligase (Mkrn1) expressions were upregulated in the infarcted areas.

We further determined the expression of genes related to macroautophagy and mitophagy in both infarcted and non‐infarcted zones following MIR injury. The result showed a significant reduction in macroautophagy‐related gene expression (Figure 6A–E), while the expression of mitophagy‐related genes (BNIP3l and Pink1) was increased within the infarcted areas (Figure 6F–H).

We also detected a significant increase in ubiquitin (Ubb) and makorin ring finger protein 1 (MKRN1) expression in the infarct areas (Figure 6I,J). Collectively, these data strongly suggested that deficiency in macroautophagy and excessive mitophagy through ubiquitin‐dependent and non‐ubiquitin‐dependent protein degradation may aggravate MIR injury.

### 2.8 Integration of Single‐Cell and Spatial Transcriptomics Reveals Dynamic Activation of Piezo1 After MIR

**FIGURE 7:**
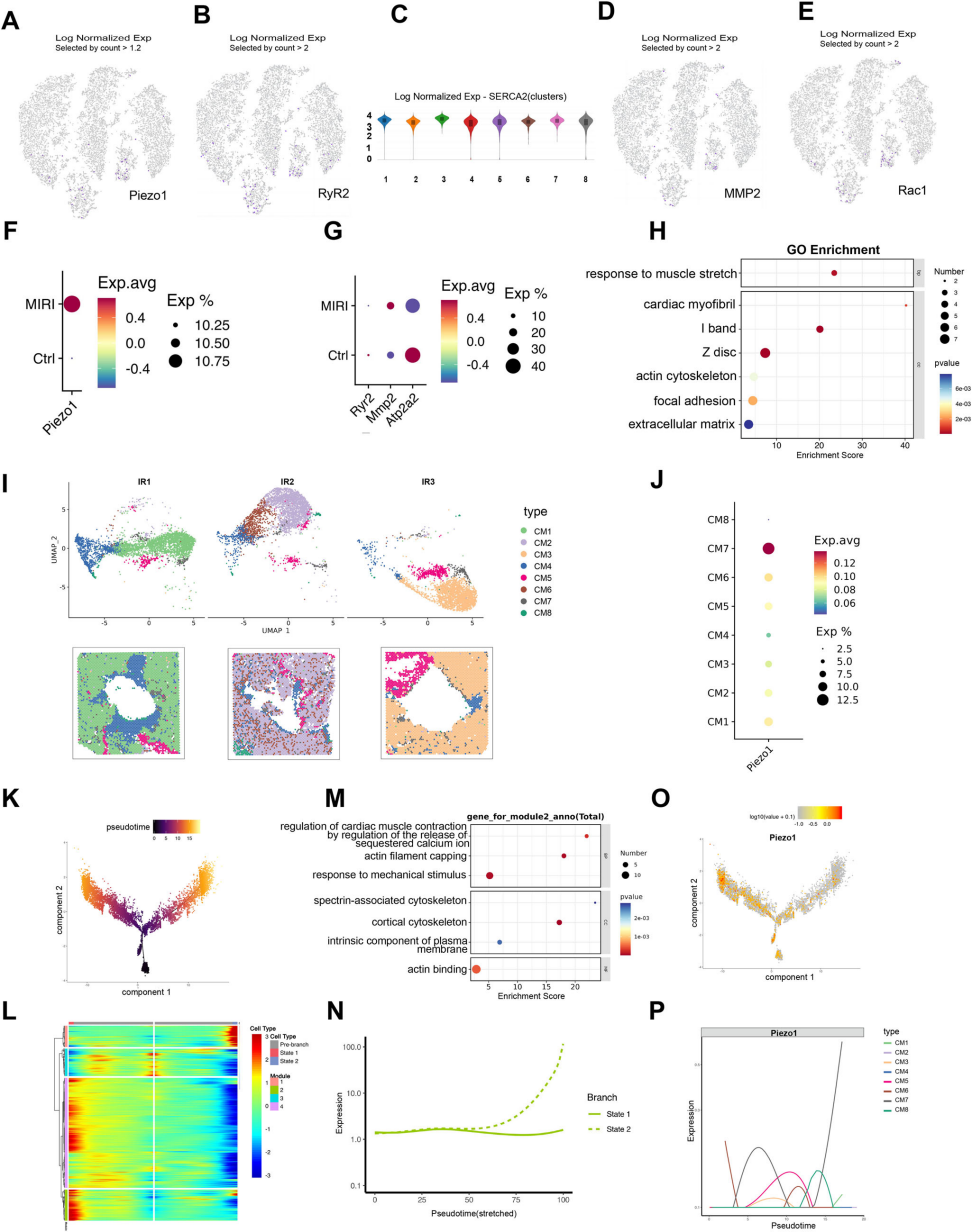
Integration of single‐cell and spatial transcriptomics reveals dynamic activation of Piezo1 after MIR. (A–E) The gene expression of Piezo1, RyR2, MMP2, and Rac1 was specifically upregulated, whereas the SERCA2 was downregulated in the infarcted and incomplete infarct zones. (F and G) Dot plots comparing expression patterns of Piezo1, Ryr2, Mmp2, and Atp2a2 between MIR and control sections. Dot color represents average expression (Exp. avg), and dot size represents the proportion of expressing spots (Exp%). (H) Gene Ontology (GO) enrichment analysis of upregulated genes in the MIRI group. (I) Integration of spatial transcriptomics and scRNA‐seq using RCTD, which mapped cardiomyocytes into eight subclusters (CM1–CM8), visualized by UMAP (top) and projected onto Visium sections (bottom). (J) Dot plot of Piezo1 expression across CM1–CM8. (K) Pseudotime trajectory of cardiomyocytes, color denotes pseudotime rank along the inferred manifold. (L) Heatmap of trajectory‐associated gene modules ordered by pseudotime, stratified by pre‐branch and branch states. (M) GO enrichment results for Module 2. (N) Expression of Module 2 along the two branch states across pseudotime. (O) Feature map of Piezo1 expression over the pseudotime manifold, color denotes log‐normalized expression. (P) Piezo1 expression along pseudotime for CM1–CM8, showing subcluster‐specific dynamics.

Spatial analysis showed that the gene expression of Piezo1, RyR2, MMP2, and Rac1 was specifically upregulated, whereas the SERCA2 was downregulated in the infarcted and incomplete infarct zones (Figure 7A–E). To further verify these findings, we analyzed single‐cell RNA sequencing data and found that Piezo1, MMP2, and SERCA2 (Atp2a2 in scRNA‐seq annotation) were elevated in the MIR group compared with the sham group, whereas RyR2 did not show a significant change (Figure 7F,G). This discrepancy between transcriptomic and protein levels may reflect post‐transcriptional regulation or differences in detection sensitivity. GO enrichment analysis was conducted in the MIR group to examine the functional significance of the upregulated genes (Figure 7H). The results revealed significant enrichment in terms related to muscle stretch response, sarcomeric structures (cardiac myofibril, I band, and Z disc), actin cytoskeleton organization, focal adhesion, and extracellular matrix, suggesting that mechanical transduction and structural remodeling are prominently involved in the injured myocardium.

Next, we applied RCTD to map the single‐cell transcriptome onto the spatial data and identified eight cardiomyocyte subclusters, as shown in Figure 7I. Notably, Piezo1 expression was predominantly enriched in CM7 (Figure 7J), implicating this subcluster as a key cellular source of Piezo1‐mediated signaling. Based on this, we then performed pseudotime trajectory analysis on cardiomyocytes and identified five cell states (States 1–5), with State 4 defined as the root of the trajectory. From this root, the trajectory bifurcated into two major branches extending toward distinct terminal states (Figure S10, Figure 7K).

Further analysis identified four gene expression modules along the trajectory (Figure 7L), among which Module 2 was significantly enriched in terms associated with calcium‐mediated regulation of cardiac contraction, response to mechanical stimulus, and cytoskeletal organization (Figure 7M). Notably, Module 2 exhibited a progressively increasing trend toward one terminal state (Figure 7N). In parallel, Piezo1 expression also showed a gradual upregulation along pseudotime, with the most prominent increase observed in CM7 (Figure 7O,P).

In summary, the spatial transcriptomic and pseudotime analyses consistently demonstrated that Piezo1 was predominantly activated in CM7 cardiomyocytes following MIR, with its expression showing a progressive increase along the course of injury. Together with the GO enrichment analysis, these findings suggest that Piezo1 may be involved in mechanotransduction and cytoskeletal remodeling processes and might contribute to calcium regulation in the injured myocardium.

### 2.9 Piezo1‐Mediated Calcium Overload Involving the MMP2–RyR2/SERCA2 Axis During Myocardial Ischemia Reperfusion Injury

**FIGURE 8:**
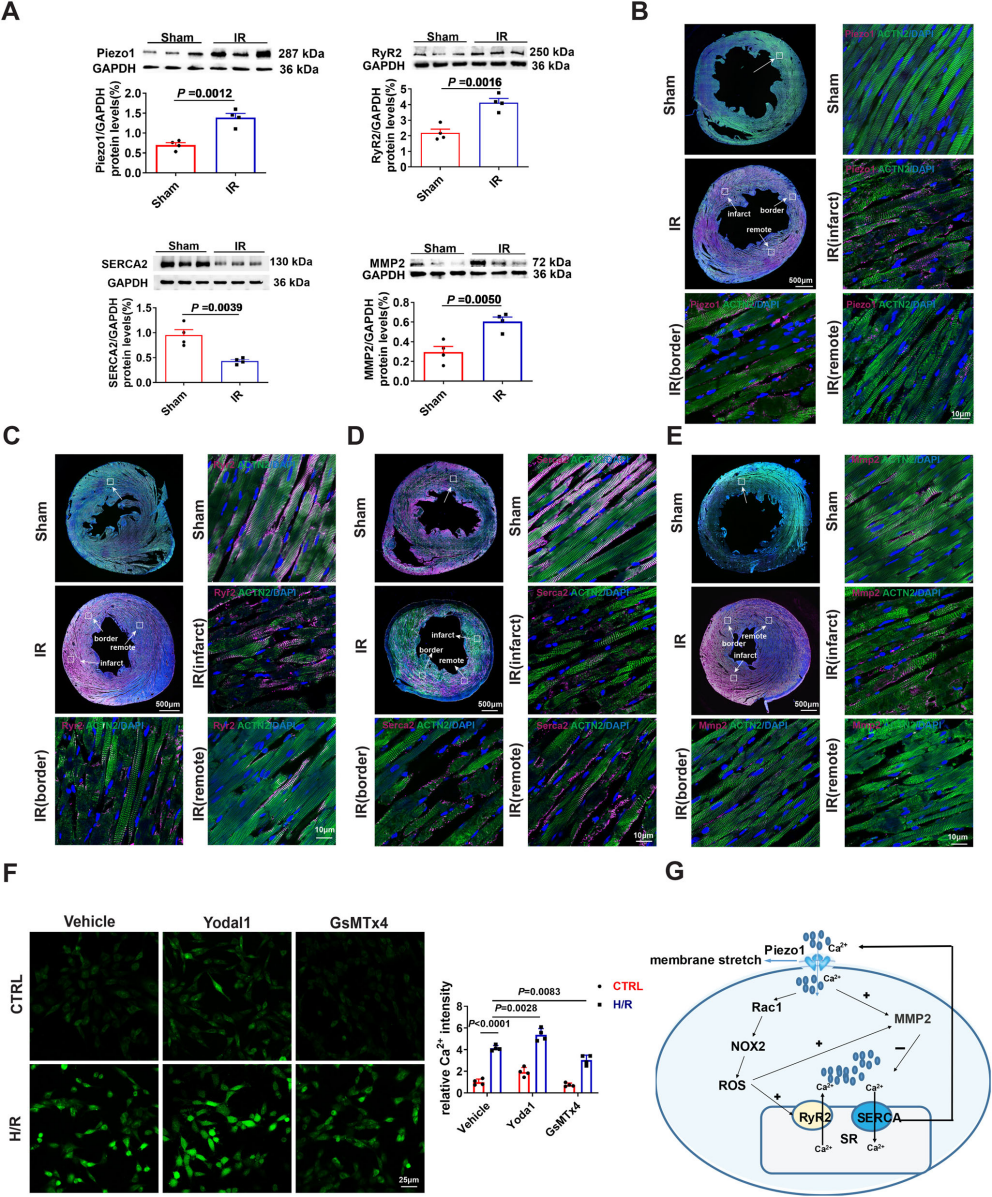
Piezo1 may mediate myocardial ischemia‐reperfusion injury through activation of MMP2–RyR2/SERCA2 signaling. (A) Representative Western blots and quantification showing protein levels of Piezo1, RyR2, SERCA2, and MMP2 proteins in the left ventricle from rats that underwent IR (30 min of ischemia followed by 2 h of reperfusion) or Sham. Sham and I/R group samples were obtained from separate cohorts of four rats each, with I/R samples collected distal to the ligation site. Each target band was first normalized with GAPDH, and then the fold change was calculated versus the Sham group (*n* = 4). (B–E) Representative immunofluorescence images showing expression of Piezo1 (B), RyR2 (C), SERCA2 (D), and MMP2 (E) (magenta) co‐stained with ACTN2 (green) in sections from another set of Sham rats and I/R rats, with the latter obtained distal to the ligation site. Scale bars = 10 µm (*n* = 4). (F) Ca^2+^ fluorescent staining with Fluo‐4 AM in H9C2 cells (left) and quantification of the Ca^2+^ fluorescence intensity (right, *n* = 4). (G) Potential model indicating Piezo1 mediation of myocardial ischemia‐reperfusion injury through activation of MMP2–RyR2/SERCA2 signaling.

We used Western blot and immunofluorescence to further validate these genes for Piezo1‐mediated calcium overload associated with MI/R injury. Western blot analysis revealed that Piezo1, RyR2, and MMP2 were significantly upregulated, whereas SERCA2 was downregulated in rats after myocardial IR injury (Figure 8A). Immunofluorescence staining further confirmed these changes, showing stronger Piezo1, RyR2, and MMP2 signals in cardiomyocytes from the infarct region compared with the border, remote, and sham groups, while SERCA2 displayed the opposite trend (Figure 8B–E). To examine the effect of Piezo1 modulation on Ca^2+^ dynamics in vitro, we subjected H9c2 cells to 8 h hypoxia followed by 2 h reoxygenation, and monitored intracellular Ca^2+^ levels using Fluo‐4 AM staining. Our measurements revealed that H/R treatment markedly increased Fluo‐4 fluorescence intensity compared with control cells. Notably, treatment with the Piezo1 inhibitor significantly attenuated the H/R‐induced Ca^2+^ elevation, whereas activation of Piezo1 enhanced Ca^2+^ influx (Figure 8F). Collectively, these data indicate that Piezo1 is associated with Ca^2+^‐handling changes involving the MMP2–RyR2/SERCA2 axis (Figure 8G). Further study into the exact mechanisms underlying Piezo1‐induced calcium overload in MIR injury is warranted.

## 3 Discussion

While a recent study has generated spatial transcriptomic maps of MIR injury in different regions of the heart using imaging mass cytometry [13], the precise spatiotemporal sequencing of molecular events underlying infarct and incomplete infarct processes remains poorly understood due to the lack of an accurate distinction between these two pathological zones. Here, we comprehensively depict the spatial landscapes of MIR injury at the translational level, focusing on precisely localized signaling pathway networks and their pathological rearrangements. Our findings not only demonstrate many similarities but also substantial differences between the processes of infarct and incomplete infarct, providing a holistic account of transcriptional heterogeneity in the microenvironment following myocardial infarction.

First, we observe distinct spatial architectures with unique functional characteristics in the border regions, incomplete infarction areas, and infarct zones. The border regions confer autophagy properties, while the infarct zones are characterized by activation of ferroptosis‐related signaling pathway genes. Interestingly, compared to both the border regions and the infarct zones, incomplete infarction areas demonstrate higher expression levels of MAPK, IL‐17, and osteoclast differentiation signaling pathway genes. This suggests a specific involvement of these signaling pathways in the processes of incomplete infarction during MIR injury. In addition to transcriptional heterogeneity at the pathway level, we also observed marked differences in the composition of immune and stromal cells across distinct regions. The infarct zones were predominantly enriched in monocytes and macrophages, accompanied by infiltration of T cells, consistent with previous studies emphasizing their central roles in orchestrating the inflammatory cascade. Previous studies have also described the dynamic recruitment of monocyte‐macrophage populations in the infarcted heart and their dual roles in amplifying inflammation and facilitating repair, while further highlighting the immunomodulatory functions of T cells, particularly regulatory T cells [28]. By contrast, the incomplete infarction areas were characterized by neutrophil infiltration together with a certain proportion of smooth muscle cells. Neutrophils have been identified as the earliest immune cells recruited during reperfusion injury, releasing proteases and reactive oxygen species that exacerbate tissue necrosis and cardiac dysfunction within a short time frame, and they preferentially accumulate in the incomplete infarction regions, thereby promoting necrotic expansion and amplifying inflammation [29, 30]. The border regions, in turn, exhibited an increased abundance of smooth muscle cells and fibroblasts, consistent with their established roles in extracellular matrix remodeling and scar formation. Previous studies have underscored the critical involvement of fibroblasts and smooth muscle cells in ECM remodeling, collagen deposition, and ventricular remodeling [31]. These region‐specific differences in cellular composition may underlie the distinct enrichment of signaling pathways observed in our study. For instance, neutrophil‐dominant areas were more prone to the activation of IL‐17 and MAPK signaling [32], whereas fibroblast and SMC‐rich regions tended to show enrichment of focal adhesion and extracellular matrix‐related pathways [33, 34].

In the incomplete infarction areas, we observed significant enrichment of the MAPK, IL‑17, and osteoclast differentiation pathways, suggesting that these signals may play central roles in regulating cardiomyocyte survival and death during the early stages of MIR injury. Previous studies have shown that IL‑17 signaling promotes cardiomyocyte apoptosis during MIR injury by inducing chemokine and cytokine expression and activating the MAPK pathway, while inhibition of MAPK by butorphanol alleviates myocardial damage [35, 36, 37, 38, 39]. Moreover, transcription factors such as Fos and Jun were markedly upregulated in these regions, consistent with previous findings indicating their involvement in macrophage activation and injury progression [27, 40]. Beyond these pathways, our KEGG enrichment analysis highlighted significant alterations in ER protein processing, proteasome‐related pathways, and focal adhesion signaling within the ischemic cardiac apex. During MIR injury, nuclear translocation of AMP‑activated protein kinase (γ2‑AMPK) alleviates ER stress and cell death by suppressing pre‑rRNA transcription and ribosome biogenesis [41]. The ER is a multifunctional signaling organelle crucial for processes such as Ca^2+^ homeostasis, protein‐folding, and lipid biosynthesis [42, 43], while excessive ER stress drives MIR injury via inflammasome and autophagy activation [42, 43, 44]. Simultaneously, the ubiquitin‐proteasome system plays a vital role in maintaining protein homeostasis by degrading damaged intracellular proteins [44, 45]; its dysfunction exacerbates injury, and proteasome inhibition abolishes the cardioprotective effect of *N*‑arachidonoylphenolamine by enhancing oxidative stress, apoptosis, and necroptosis [45, 46, 47]. Furthermore, focal adhesion kinase (FAK), a tyrosine kinase essential for focal adhesion assembly, promotes cell survival and mitigates myocyte apoptosis through NF‑κB activation [48, 49]. Together, these findings suggest that pathways such as MAPK, IL‑17, osteoclast differentiation, as well as ER processing, proteasomal homeostasis, and focal adhesion signaling may represent potential targets for early intervention aimed at preventing further expansion of infarct size during MIR injury.

Our spatial transcriptomic data revealed that the infarct zones were significantly enriched for ferroptosis‐ and necroptosis‐related pathways, with notable upregulation of Alox15, Ftl1, and Ncoa4. These findings indicate that ferroptosis is a dominant driver of cardiomyocyte death within infarcted myocardium. Previous studies have shown that ALOX15‐induced cardiomyocyte ferroptosis is driven by mitochondrial damage [50]. Considering that ferroptosis is driven by iron overload, ferritinophagy has been identified as a key contributor [50, 51]. Ferritin degradation within the autophagosome, mediated by Ncoa4, results in free iron release, and deletion of Ncoa4 improves cardiac function [52]. Consistent with this, our analysis showed that Ncoa4 was specifically upregulated in infarct zones. By contrast, HMOX‐1 was elevated in incomplete infarct regions. Previous studies have reported that HMOX‐1 mediates heme degradation and Fe^2^⁺ release, thereby contributing to iron overload and ferroptosis [53], whereas HMOX‐1 overexpression can mitigate ferroptosis by upregulating SLC7A11 (a component of systemXc−), thereby limiting I/R injury [54]. In contrast, TFRC and USP7 showed no significant changes between infarct and non‐infarct regions. Previous studies have reported that TFRC promotes ferroptosis during MIR injury by mediating Fe^3^⁺ uptake, and its activity can be positively regulated by deubiquitination through USP7 [51, 55]. The absence of expression changes in our dataset, therefore, suggests that ferritinophagy, rather than transferrin transport, may represent the predominant source of iron overload in this context. GPX4 axis is considered the primary pathway for preventing ferroptosis [50] and may be downstream of Alox15 [56]. However, we observed no significant differences among the groups. In addition to iron‐handling pathways, we also noted alterations in stress response chaperones. Interestingly, heat shock protein 70 (HSP70), particularly Hspa1b and Hsph1, exhibited downregulation in the infarcted zones, but upregulation exclusively in the incomplete infarct areas. Given that HSP70 family proteins promote correct protein folding, reduce ROS accumulation, and stabilize calcium homeostasis, these patterns are consistent with protection in partially preserved regions and loss of chaperone support in infarct areas [57, 58].

Beyond ferroptosis, we observed marked dysregulation of autophagic processes within infarct zones: macroautophagy‐related genes were reduced, while mitophagy‐related genes (BNIP3l, Pink1) were increased, together with elevated Ubb and MKRN1. Mitophagy is a mechanism that selectively degrades damaged mitochondria to suppress ferroptosis through autophagic flux, occurring via ubiquitin‐dependent and ubiquitin‐independent pathways [59]. Prior studies suggest that mitophagy can play dual roles, as moderate flux may limit ROS and iron availability, while excessive mitophagy may release additional iron and amplify lipid peroxidation and ferroptosis [59]. Herein, we identified upregulation of genes involved in both ubiquitin‐dependent and non‐ubiquitin‐dependent protein degradation pathways in the infarcted zones following MIR injury. Additionally, we observed an increase in the expression of genes associated with ferroptosis and mitophagy in these regions. These findings are consistent with previous reports highlighting the detrimental effects of excessive mitophagy and ferroptosis on MIR injury [50, 60]. Therefore, targeting ferroptosis by rescuing mitophagy may represent a promising therapeutic strategy for safeguarding cardiomyocytes against MIR injury.

Myocardial ischemia‐reperfusion can increase the myocardium's susceptibility to dangerous arrhythmias, such as ventricular tachycardias and fibrillation [61]. Trdn (Triadin), a part of the calcium release complex, plays an essential role in skeletal muscle excitation–contraction coupling in the cardiomyocytes as well as regulating the rate of heartbeats [62]. The deficiency of triadin in mice has been confirmed to exacerbate Ca^2+^ overload and impair the contractile recovery following MIR injury [63]. In people, TRDN mutations are linked to a genetic form of ventricular arrhythmia [62], and ablation of TRDN renders the hearts susceptible to ventricular arrhythmias [63]. Previous studies have shown that Trdn null mice exhibit a significantly higher incidence of ventricular arrhythmias upon β‐adrenergic stimulation compared to Trdn^+/+^ mice [64]. We observed an effective suppression of TRDN levels in infarcted zones following MIR injury, providing direct evidence supporting the recommendation for long‐term use of β‐blockers to improve survival in patients recovering from acute myocardial infarction [65]. This result also suggests that targeting TRDN could be a viable approach for reducing the rate of ventricular arrhythmias during MIR injury.

Calcium overload is a vital mechanism of MIR injury. Our spatial transcriptomic and single‐cell analyses revealed that Piezo1, RyR2, and MMP2 were consistently upregulated, whereas SERCA2 was downregulated in both infarct and incomplete infarct zones. These changes were validated by Western blot and immunofluorescence, and further confirmed in vitro, where modulation of Piezo1 significantly altered H/R‐induced Ca^2+^ influx. Together, these data highlight Piezo1 as a key mechanosensor associated with calcium‐handling alterations during MIR injury. Mechanosensitive Piezo1 cation channel is characteristically activated by multiform mechanical stimulation, and has been implicated in initiating Ca^2+^ influx‐Rac1‐NOX2‐ROS‐Ca^2+^ spark signaling pathway [19, 66]. Studies have shown that Piezo1‐mediated neurogenic inflammatory cascade exacerbates ventricular remodeling after myocardial infarction [67]. Ryanodine receptor 2 (RyR2), a ryanodine receptor on the sarcoplasmic reticulum, normally mediates Ca^2+^ release to support excitation–contraction coupling, but becomes hyperactive under oxidative stress, thereby amplifying Ca^2+^ overload [19, 68]. Overexpression of sarcoplasmic/endoplasmic reticulum Ca^2+^‐ATPase2 (SERCA2), by contrast, attenuates Ca^2+^ overload and protects cardiac microcirculation against MI/R injury by sustaining the mitochondrial quality control [69]. Notably, SERCA2 has been shown to inhibit Piezo1‐mediated mechanosensitive currents [70], and the activation of myocardial matrix metalloproteinase2 (MMP2) by ROS contributes to SERCA2 proteolysis during myocardial IR injury [71]. Collectively, these results indicate that Piezo1 upregulation is associated with Ca^2+^‐handling changes involving the MMP2–RyR2/SERCA2 axis during MIR injury.

This study has several limitations. The spatial transcriptomic profiling was performed without the inclusion of an independent sham‐operated control group. Instead, an internal reference framework was adopted, in which infarct zones, incomplete infarction areas, border regions, and anatomically preserved non‐infarcted regions within the same heart were compared. In addition, tissue sampling was performed at a single reperfusion time point, which limits the ability to capture the temporal dynamics of MIR injury. At the same time, the 10x Visium platform has limited spatial resolution, and the RCTD‐based deconvolution represents a probabilistic inference that depends on the single‐cell reference and model assumptions; therefore, our findings focus on regional patterns and major cell‐type trends. Future studies incorporating sham‐operated controls and sampling across multiple reperfusion intervals will be important to further validate and extend the present findings.

## 4 Conclusion

In conclusion, our study provides detailed insight into the transcriptional heterogeneity in the infarcted regions, incomplete infarct areas, and their neighboring areas. By integrating spatial transcriptomics with additional single‐cell sequencing, we were able to validate Piezo1‐associated alterations and highlight their roles in calcium‐handling dysregulation. Together, these results provide a framework for future mechanistic studies and suggest potential therapeutic strategies for early intervention to prevent infarct size expansion and rescue cardiomyocyte death during MIR injury.

## 5 Materials and Methods

### 5.1 Experimental Animals

The male Sprague–Dawley rats aged 9 weeks were obtained from the Institute of Laboratory Animal Science at Tongji Medical College, Huazhong University of Science and Technology. They were housed under controlled environmental conditions (temperature ranging from 22°C to 25°C, with a 12‐h light/dark cycle) and provided ad libitum access to food and water.

### 5.2 Rats Myocardial Ischemia‐Reperfusion Model

The MIR injury model was established by performing left anterior descending coronary artery ligation surgery, following the previously described protocol with minor modifications [67, 72]. Initially, the rats were anesthetized using pentobarbital sodium and then mechanically ventilated after endotracheal intubation with a 14‐gauge angiocatheter. Heart function was monitored using a rat electrocardiogram (ECG) system. Subsequently, a thoracotomy was performed at the left fourth intercostal space to ligate the heart around the left anterior descending artery, specifically 2 mm below the left atrium, using 6‐0 Polypropylene sutures. After 30 min of occlusion, myocardial reperfusion was allowed for 2 h by removing the ligation. Ischemia presence was confirmed through ST‐segment elevation in ECG and visible color changes in the ischemic region of myocardial tissue.

### 5.3 Sample Preparation and Imaging

Heart tissues were obtained from three rats subjected to myocardial ischemia‐reperfusion. From each heart, a short‐axis transverse section was collected at a level below the site of coronary artery ligation. These sections encompassed the full ventricular cross‐section, including the infarcted region of the left ventricle and the surrounding peri‐infarct tissue. These sections were meticulously cleansed of any blood stains using sterile wipes. The prepared tissues were then embedded in OCT, frozen at −80°C, and subsequently sectioned into 10 µm thick slices. These slices were mounted on spatial transcriptomics arrays, dehydrated briefly in isopropanol, stained with H&E, mounted in 80% glycerol, and imaged using a high‐resolution 3D HISTECH Pannoramic MIDI FL scanner at 40× magnification. The slides for library preparation were obtained from the Spatial Transcriptomics team (available at https://www.10xgenomics.com/). Each spot on the array is 55 µm in diameter, spaced 100 µm apart, covering an area of approximately 6.5 × 6.5 mm^2^. Each slide consists of four capture areas containing around 5000 distinct gene expression spots within each area.

### 5.4 Tissue Optimization

These tissue samples are precisely placed onto designated capture areas of the Visium Spatial Tissue Optimization Slide. These sections undergo fixation, staining, and permeabilization processes for specific durations. During the permeabilization process, the mRNA released binds to the capture probes on the slide. Subsequently, cDNA is generated using fluorescently labeled nucleotides to enable visualization of the synthesized cDNA. Finally, enzymatic removal of the tissue reveals the presence of fluorescently labeled cDNA. This labeled cDNA can be observed under fluorescence microscopy to determine the optimal duration for permeabilization. The optimal duration is achieved when a maximum fluorescence signal is obtained with minimal signal diffusion. In cases where equivalent signal intensity is observed at two distinct time points, a longer duration for permeabilization is considered preferable.

### 5.5 cDNA Synthesis, Library Construction, and Sequencing

Tissue sections are placed onto capture areas where they undergo fixation, staining, and permeabilization. Subsequently, cellular mRNA is captured by primers located on gene expression spots that generate unique spatial barcodes for each cDNA molecule. Libraries are constructed from these cDNAs and sequenced using the NovaSeq 6000 System (Illumina Inc., San Diego, CA, USA) with a minimum sequencing depth of 50,000 read pairs achieved for each barcode region. The spatial barcodes facilitate the association of sequencing reads back to tissue section images for spatial gene expression mapping.

### 5.6 **Single‐Cell RNA Sequencin**g and Integration Analysis

Left ventricular samples were derived from sham and MIR rat hearts, with one sample analyzed per condition. The infarct and peri‐infarct regions, together with anatomically matched sites from sham controls, were minced and enzymatically digested to generate single‐cell suspensions. Single‐cell libraries were prepared by OE Biotech (Shanghai, China) using a droplet‐based 3′RNA‐seq workflow (Chromium, 10x Genomics) and sequenced on an Illumina platform according to the manufacturer's instructions. Raw reads were processed to gene barcode matrices with the vendor pipeline and analyzed in Seurat using standard quality control, normalization, dimensionality reduction, clustering, and marker‐based annotation. All major cardiac cell types were retained during the initial clustering to serve as an overall reference for subsequent spatial deconvolution. In the subsequent analysis focusing on Piezo1, cardiomyocytes were selectively extracted and combined with pseudotime analysis to assess their dynamic changes. Piezo1 expression was quantified per cell from log‐normalized counts, and sham versus MIR differences were tested with two‐sided Wilcoxon rank‐sum tests with Benjamini–Hochberg correction. Cardiomyocyte subclusters obtained by reclustering served as the reference in RCTD to deconvolve Visium spots and map cardiomyocyte states in space. Pseudotime was inferred with Monocle, rooting the trajectory in sham‐enriched states, and Piezo1 was examined as a function of pseudotime along the inferred continuum.

### 5.7 Western Blotting

The heart tissues were homogenized in ice‐cold RIPA buffer and lysed at 4°C for 30 min. After centrifugation of the lysate at 12,000 × *g* at 4°C for 10 min, the supernatants containing proteins were quantified using the BCA Protein Assay Kit (AR1189, Boster). The protein was then separated by 8%–10% sodium dodecyl sulfate‐polyacrylamide gel electrophoresis and subsequently transferred to PVDF membranes. The membranes were blocked with 5% nonfat milk in TBST (Tris‐buffered saline with 0.1% Tween 20) at room temperature for 1 h. Subsequently, the membranes were incubated overnight at 4°C with the primary antibodies against Piezo1 (1:1000, A4340, ABclonal), Ryr2 (1:1000, 19765‐1‐AP, Proteintech), SERCA2 (1:1000, ab150453, Abcam), and MMP2 (1:1000, ab92536, Abcam). After washing in TBST, the protein bands on membranes were visualized using ECL Western blotting detection reagents (Vazyme) on Bio‐Rad ChemiDoc Touch and further quantified using ImageJ software.

### 5.8 Immunofluorescence Staining

The heart tissues of rats were fixed in 4% paraformaldehyde at room temperature and then serially sectioned at a thickness of 40 µm. After being washed with 1×PBS, the sections were blocked with 10% goat serum and 0.3% Triton X‐100. Subsequently, the heart sections were incubated with primary antibodies, including anti‐ACTN2 (1:200, A7811, Sigma‐Aldrich), anti‐Piezo1 (1:100, A4340, ABclonal), anti‐Ryr2 (1:100, 19765‐1‐AP, Proteintech), anti‐SERCA2 (1:100, ab150453, Abcam), or anti‐MMP2 (1:100, ab92536, Abcam). The primary antibody at 4°C overnight and 1:400 for secondary antibody was used at room temperature for 1.5 h. Finally, DAPI staining was used to visualize the nuclei. All immunofluorescence images were captured using a Leica SP8 confocal microscope from Leica Microsystems in Germany.

### 5.9 Cell Culture and Protocols

Rat cardiomyoblast H9c2 cells (Procell Life Science & Technology Co. Ltd.) were cultured in Dulbecco's modified Eagle's medium (DMEM, Gibco) supplemented with 10% fetal bovine serum in a humidified incubator at 37°C with 5% CO_2_. When cultures reached 70%–80% confluence, hypoxia–reoxygenation (H/R) experiments were performed. Cells were switched to glucose‐free DMEM (Shanghai BasalMedia Technologies Co. Ltd.) and placed in an anaerobic chamber (95% N_2_/5% CO_2_) at 37°C for 8 h; during hypoxia, cells were concurrently treated with the Piezo1 inhibitor GsMTx4 (5 µM, Y241608, Beyotime) and the agonist Yoda1 (5 µM, Y239559, Beyotime). Subsequently, cells were returned to complete medium and reoxygenated under normoxic conditions (95% O_2_/5% CO_2_) at 37°C for 2 h, with GsMTx4 and Yoda1 administered again at the onset of reoxygenation.

### 5.10 Intracellular Ca^2+^ Measurements

Following the previously described method [73], H9c2 cells were loaded with 1 µM Fluo‐4 AM (S1060, Beyotime) in HBSS at 37°C for 30 min. Fluorescence was recorded with a laser‐scanning confocal microscope, with 488 nm excitation and emission collected at 516 nm. Five to six non‐overlapping fields were sampled at random, and mean fluorescence intensity was quantified in ImageJ.

### 5.11 Data Analysis

The raw FASTQ files were processed and aligned to the mRatBN7.2 reference genome using 10× Genomics' Space Ranger software (version 2.0.1). This involved summarizing unique molecular identifier (UMI) counts for each barcode. Subsequently, tissue overlaying spots were distinguished from the background through imaging analysis. The filtered UMI count matrix was then analyzed using the Seurat R package (v4.1.0). Data normalization and identification of the top 3000 highly variable genes were facilitated by Sctransform. Principal component analysis (PCA) was employed to simplify the log‐transformed gene‐barcode matrices.

The genes were visualized with a two‐dimensional uniform manifold approximation and projection (UMAP) using the RunUMAP function. The FindAllMarkers function was utilized to identify marker genes for each cluster and select differentially expressed genes (DEGs). A statistically significant threshold for differential gene expression was set, with a p‐value less than 0.05 and an absolute log2 fold change greater than 0.58. The adjusted *p*‐value was based on Bonferroni correction using all genes in the dataset. Gene Ontology (GO) and Kyoto Encyclopedia of Genes and Genomes (KEGG) pathway enrichment analyses were performed on the DEGs using R (version 4.0.3) based on the hypergeometric distribution.

To integrate spatial transcriptomics with single‐cell data, robust cell type decomposition (RCTD) was applied, enabling the mapping of cardiomyocyte subclusters onto spatial coordinates. Furthermore, pseudotime trajectory analysis was conducted using Monocle2 to order cardiomyocytes along dynamic states, identify gene expression modules, and assess their functional enrichment. OE Biotech Co. Ltd. (Shanghai, China) provided sequencing services and bioinformatics analysis.

## Author Contributions

Zhen Li, Duozhi Wu, and Hongbing Xiang conceived and designed the study. Zhen Li, Fan Jiang, and Yan Chen performed most experiments and analyzed data. Zhixiao Li, Yanqiong Wu, and Zhigang He performed the experiments and data analysis. Zhen Li wrote the original draft of the manuscript. Fan Jiang revised the manuscript and contributed to the additional experiments. Duozhi Wu and Hongbing Xiang revised the manuscript. All of the authors have read and approved the final version of the manuscript.

## Funding

This project was supported by the National Natural Science Foundation of China (Nos. 82401466; 81873467), China Postdoctoral Science Foundation (2024M761044), and the Hainan Province Clinical Medical Center and the Key Research and Development Program of Hainan Province (ZDYF2021SHFZ087).

## Ethics Statement

The entire experimental protocol was reviewed and approved by the Institutional Animal Care and Use Committee of Tongji Hospital, Tongji Medical College, Huazhong University of Science and Technology, Wuhan, China, with ethical approval number TJ‐A0803.

## Conflicts of Interest

The authors declare no conflicts of interest.

## Supporting information




**Figure S1–S8**: Marker gene expression for Louvain Clusters 1–8. Representative marker genes of Louvain clusters (Clusters 1–8) are shown in UMAP and spatial feature plots.
**Figure S9**: KEGG metabolic pathway enrichment analysis of marker genes in eight cardiomyocyte clusters. (A–H) Dot plots show the top 20 enriched metabolic KEGG pathways for the top 100 marker genes of each cluster. The *x*‐axis represents the enrichment score, dot size indicates the number of genes enriched in each pathway, and dot color corresponds to the adjusted *p‐*value.
**Figure S10**: KEGG pseudotime trajectory analysis of cardiomyocytes. (A) Pseudotime trajectory with colors indicating cardiomyocyte subcluster. (B) Pseudotime trajectory color‐coded by cell states (States 1–5), with State 4 defined as the root of the trajectory. (C) Pie charts showing the distribution of cell states within each cardiomyocyte subcluster. (D) Pie charts showing the distribution of cell states across different experimental groups (IR1, IR2, IR3).

## Data Availability

The raw spatial transcriptomics data generated in this study have been deposited in the NGDC‐CROST under accession number PRJCA045822. All other data supporting the findings of this study are available from the corresponding authors upon reasonable request. In addition, the custom code used for spatial transcriptomic analysis has been uploaded to GitHub at DOI https://github.com/yujhao/single_cell_RNA‐seq/tree/main.
