# Supplementary material for: Spatial Transcriptional Heterogeneity in the Infarct Core and Its Surrounding Regions Targeting Piezo1 Signals in Rats With Myocardial Ischemia‐Reperfusion Injury
Source: MedComm (2020). 2026 Jan 2;7(1):e70537. doi: 10.1002/mco2.70537 (PMC12757850; doi:10.1002/mco2.70537)
Supplement: Supplementary file 1 — Figure S1–S8: Marker gene expression for Louvain Clusters 1–8. Representative marker genes of Louvain clusters (Clusters 1–8) are shown in UMAP and spatial feature plots. Figure S9: KEGG metabolic pathway enrichment analysis of marker genes in eight cardiomyocyte clusters. (A–H) Dot plots show the top 20 enriched metabolic KEGG pathways for the top 100 marker genes of each cluster. The x‐axis represents the enrichment score, dot size indicates the number of genes enriched in each pathway, and dot color corresponds to the adjusted p‐value. Figure S10: KEGG pseudotime trajectory analysis of cardiomyocytes. (A) Pseudotime trajectory with colors indicating cardiomyocyte subcluster. (B) Pseudotime trajectory color‐coded by cell states (States 1–5), with State 4 defined as the root of the trajectory. (C) Pie charts showing the distribution of cell states within each cardiomyocyte subcluster. (D) Pie charts showing the distribution of cell states across different experimental groups (IR1, IR2, IR3). [file MCO2-7-e70537-s001.pdf]

Spatial transcriptional heterogeneity in the infarct core and its surrounding regions targeting Piezo1 signals in rats with myocardial ischemia-reperfusion injury

Running title: Spatial transcriptomics targeting Piezo1

Zhen Li[1#], Fan Jiang[1#], Yan chen[2#], Zhixiao Li[1], Yanqiong Wu[1], Zhigang He[1], Duozhi Wu[2*], Hongbing Xiang[1,3*]

[1] Department of Anesthesiology and Pain Medicine, Hubei Key Laboratory of Geriatric Anesthesia and Perioperative Brain Health, Wuhan Clinical Research Center for Geriatric Anesthesia, Tongji Hospital, Tongji Medical College, Huazhong University of Science and Technology, Wuhan, China;

[2] Department of Anesthesiology, Hainan General Hospital, Hainan Affiliated Hospital of Hainan Medical University, Haikou, Hainan, China;

[3] Key Laboratory of Anesthesiology and Resuscitation (Huazhong University of Science and Technology), Ministry of Education, Wuhan, China

---

[#]Zhen Li, Fan Jiang and Yan chen contributed equally to this work.

Corresponding author: Hongbing Xiang, Tongji Hospital, Tongji Medical College, No 1095, Jiefang Ave, Wuhan 430030, China. Email: hbxiang@tjh.tjmu.edu.cn;

Duozhi Wu, Department of Anesthesiology, Hainan General Hospital, Haikou 570311, Hainan, China. Email:13976674619@163.com;

Funding information

National Natural Science Foundation of China (No. 82401466; 81873467); China Postdoctoral Science Foundation (2024M761044);

Hainan Province Clinical Medical Center and the Key Research and Development Program of Hainan Province (ZDYF2021SHFZ087)

Figure S1

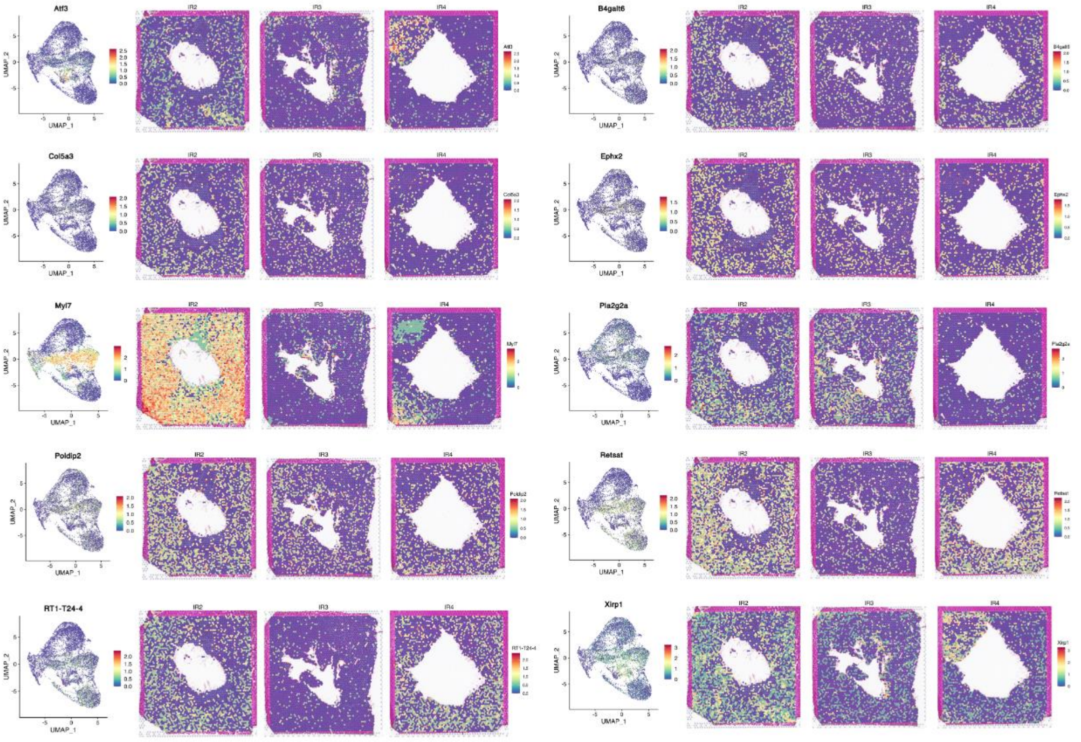

Figure S2

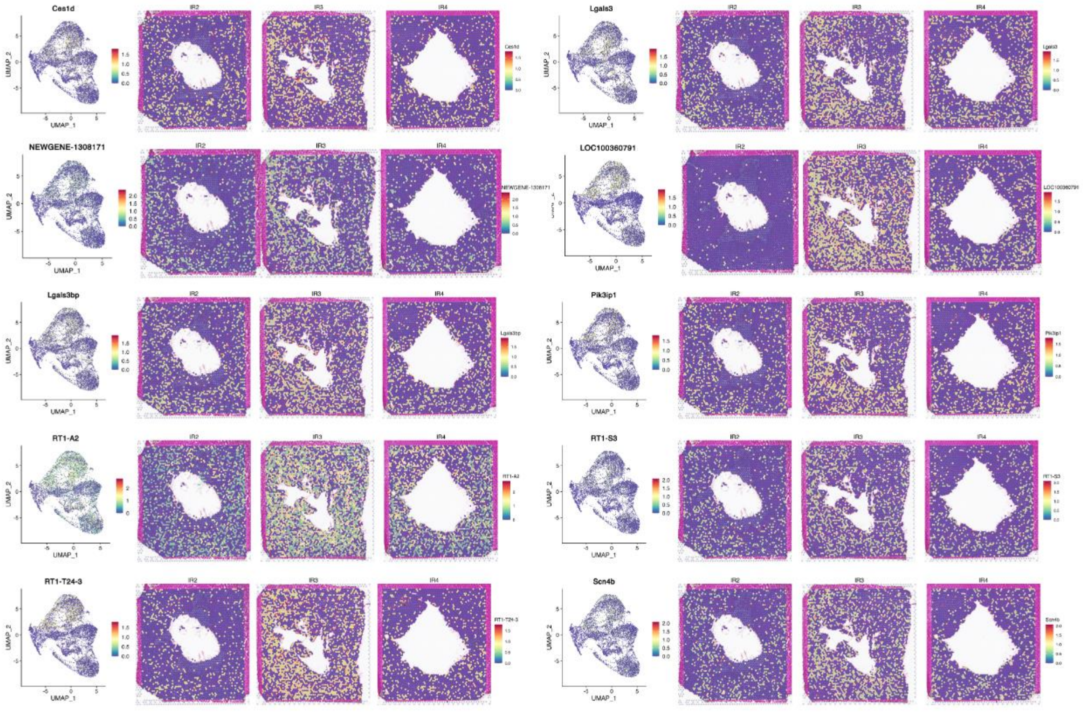

Figure S3

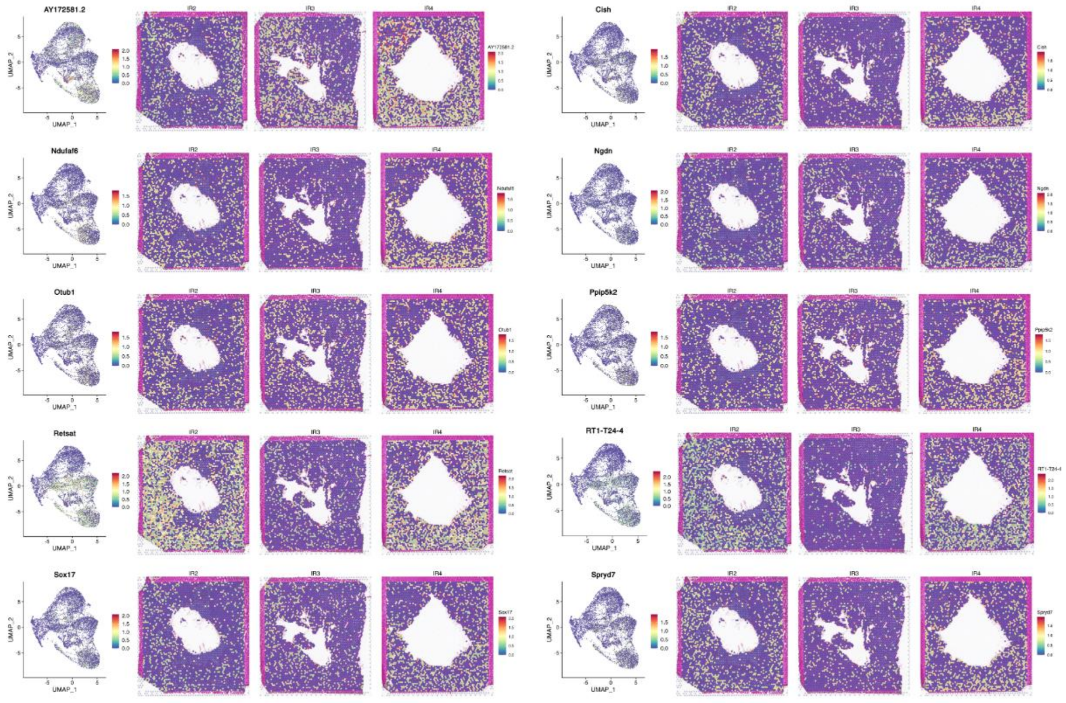

Figure S4

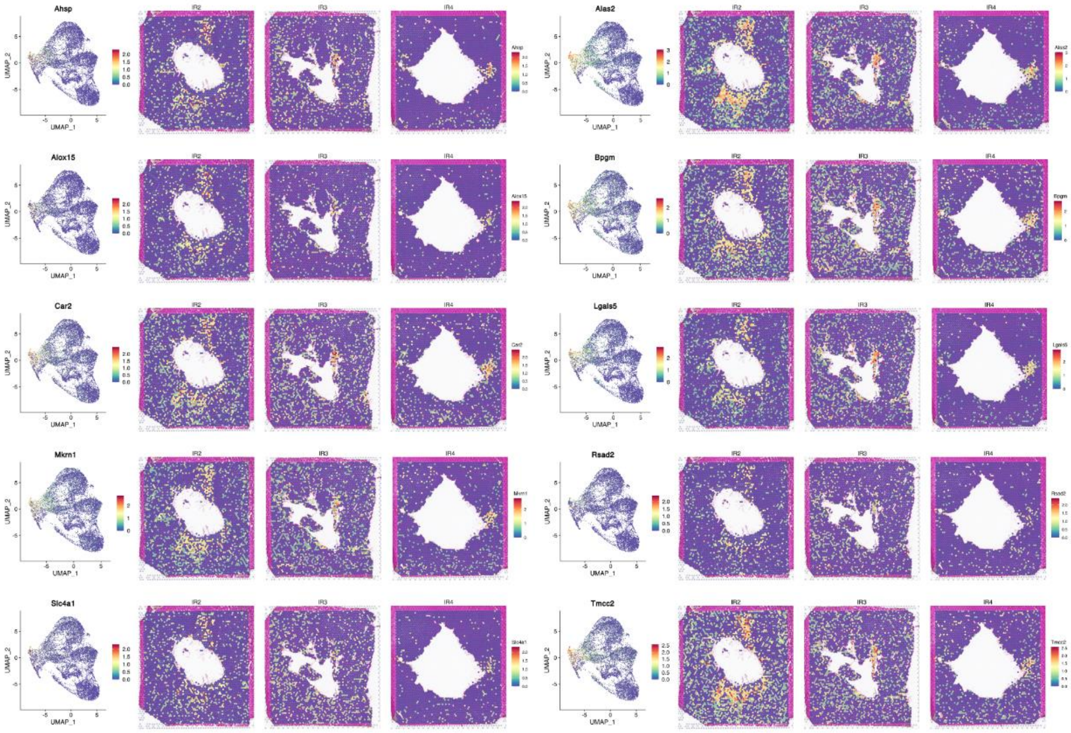

Figure S5

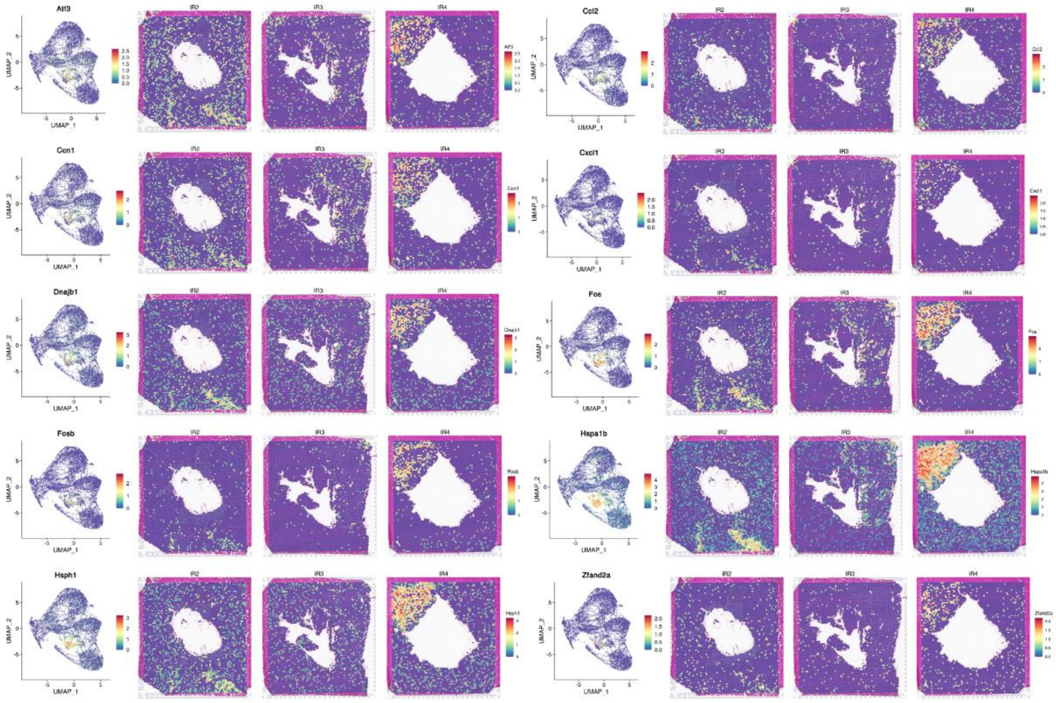

Figure S6

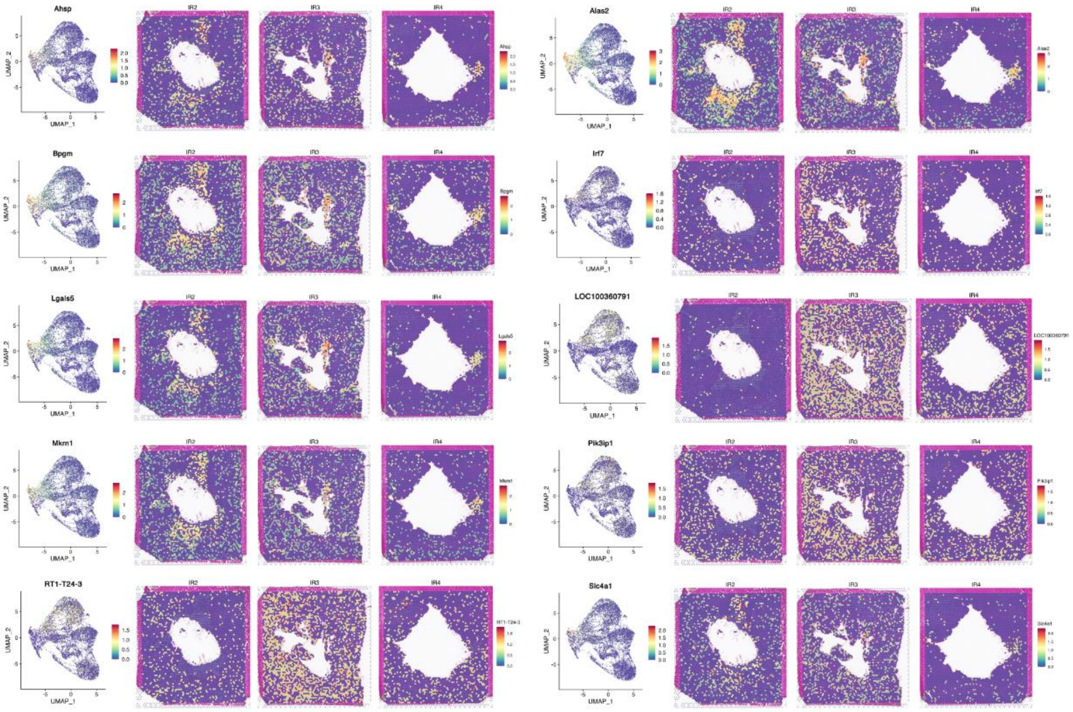

Figure S7

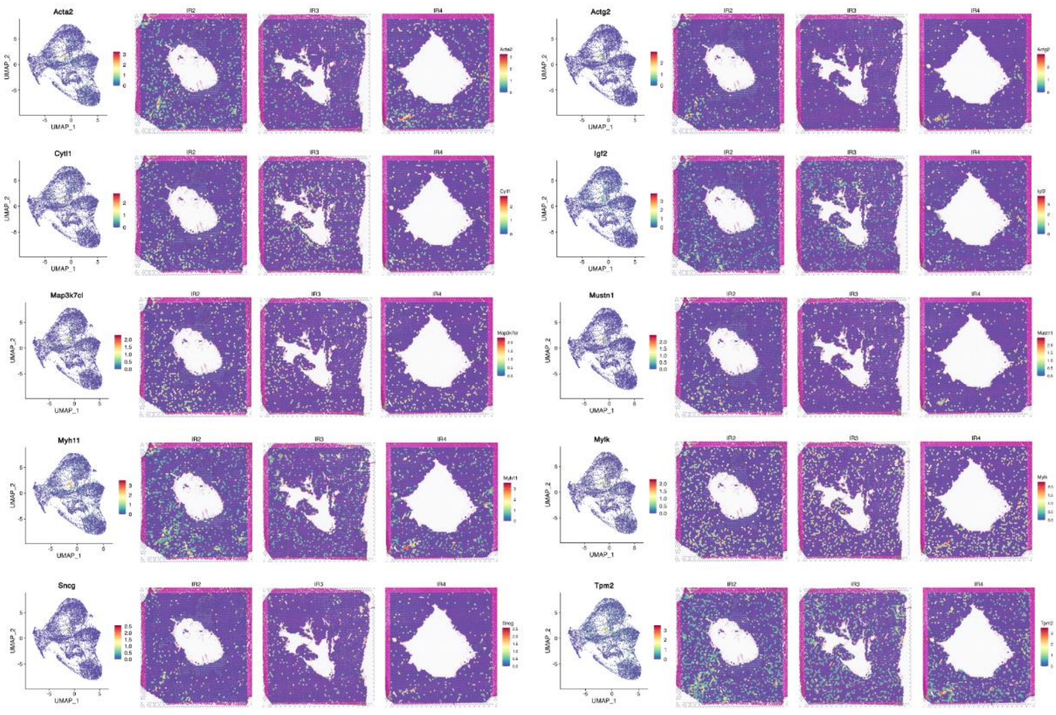

Figure S8

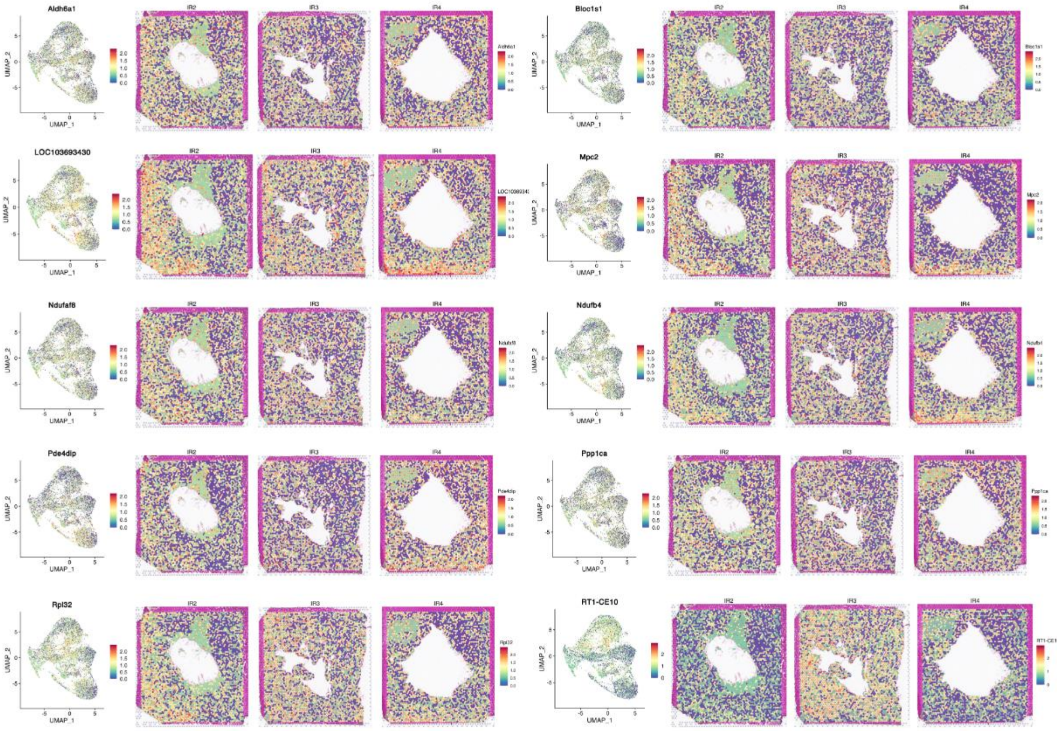

Figure S9

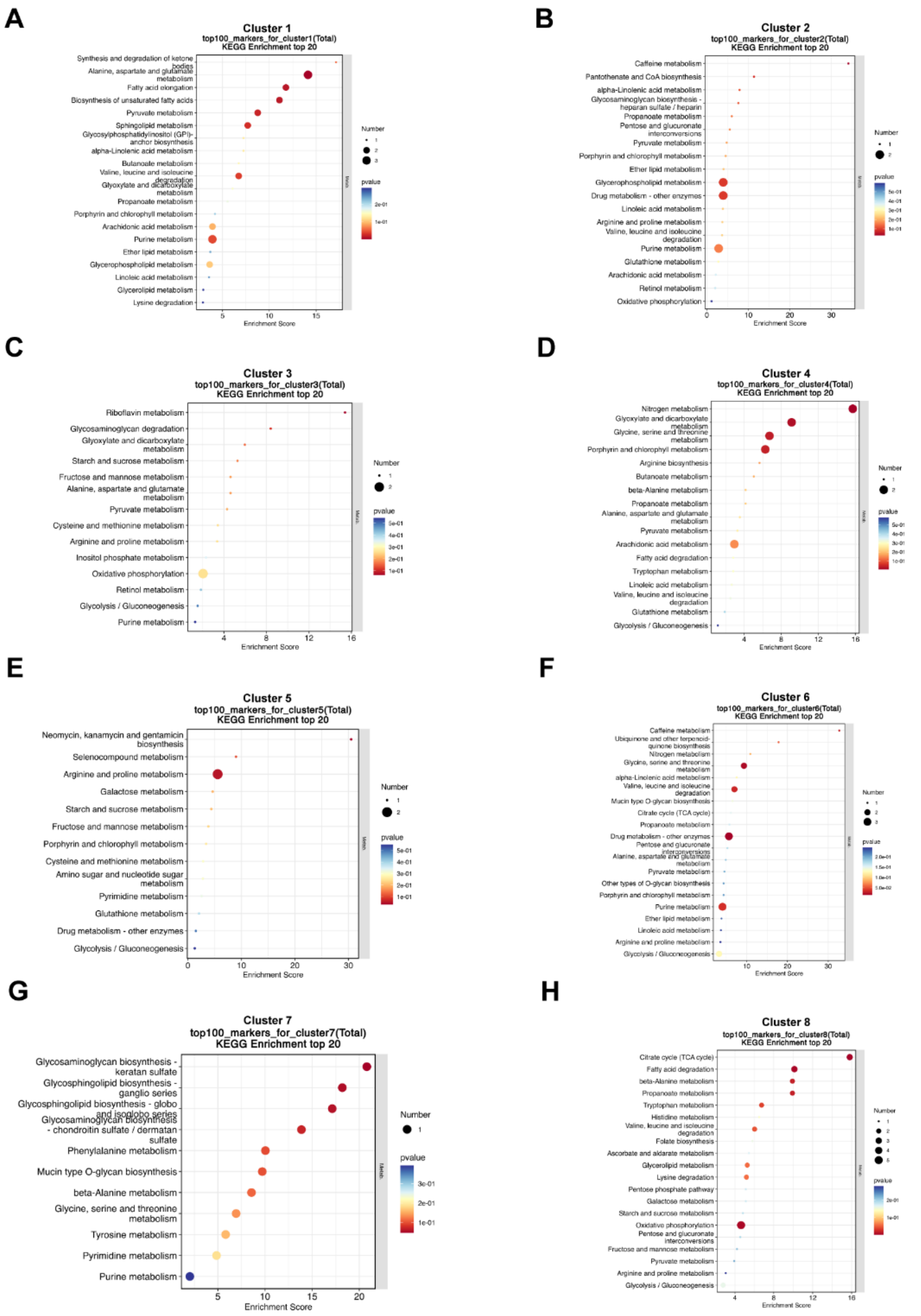

Figure S10

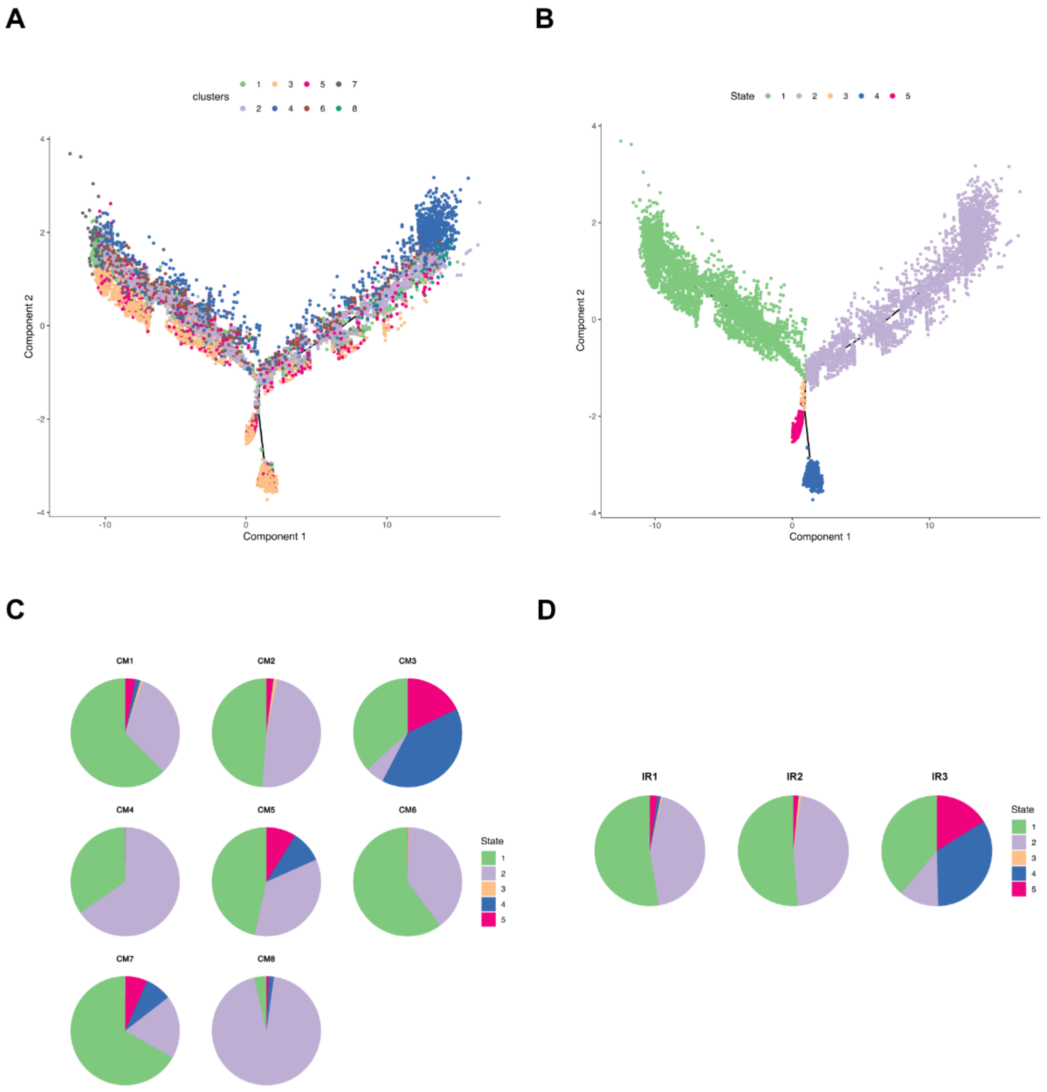

Supplemental Figure Legends

Figure S1-8 Marker gene expression for Louvain clusters 1–8.
Representative marker genes of Louvain clusters (cluster 1–8) are shown in UMAP and spatial feature plots.

Figure S9 KEGG metabolic pathway enrichment analysis of marker genes in eight cardiomyocyte clusters.
(A-H) Dot plots show the top 20 enriched metabolic KEGG pathways for the top 100 marker genes of each cluster. The x-axis represents the enrichment score, dot size indicates the number of genes enriched in each pathway, and dot color corresponds to the adjusted *P* value.

Figure S10 KEGG Pseudotime trajectory analysis of cardiomyocytes.
(A) Pseudotime trajectory with colors indicating cardiomyocyte subclusters.
(B) Pseudotime trajectory color-coded by cell states (State 1-5), with State 4 defined as the root of the trajectory.
(C) Pie charts showing the distribution of cell states within each cardiomyocyte subcluster.
(D) Pie charts showing the distribution of cell states across different experimental groups (IR1, IR2, IR3).
